# Mir-29b Mediates the Neural Tube versus Neural Crest Fate Decision during Embryonic Stem Cell Neural Differentiation

**DOI:** 10.1016/j.stemcr.2017.06.017

**Published:** 2017-07-27

**Authors:** Jiajie Xi, Yukang Wu, Guoping Li, Li Ma, Ke Feng, Xudong Guo, Wenwen Jia, Guiying Wang, Guang Yang, Ping Li, Jiuhong Kang

**Affiliations:** 1 Clinical and Translational Research Center of Shanghai First Maternity and Infant Health Hospital, Shanghai Key Laboratory of Signaling and Disease Research, School of Life Science and Technology, Tongji University, 1239 Siping Road, Shanghai 200092, PR China; 2 The Collaborative Innovation Center for Brain Science, Tongji University, Shanghai 200092, PR China

**Keywords:** ESCs, neural tube epithelial cells, neural crest cells, miR-29b, *Dnmt3a*, *Pou3f1*

## Abstract

During gastrulation, the neuroectoderm cells form the neural tube and neural crest. The nervous system contains significantly more microRNAs than other tissues, but the role of microRNAs in controlling the differentiation of neuroectodermal cells into neural tube epithelial (NTE) cells and neural crest cells (NCCs) remains unknown. Using embryonic stem cell (ESC) neural differentiation systems, we found that miR-29b was upregulated in NTE cells and downregulated in NCCs. MiR-29b promoted the differentiation of ESCs into NTE cells and inhibited their differentiation into NCCs. Accordingly, the inhibition of miR-29b significantly inhibited the differentiation of NTE cells. A mechanistic study revealed that miR-29b targets *DNA methyltransferase 3a* (*Dnmt3a*) to regulate neural differentiation. Moreover, miR-29b mediated the function of *Pou3f1*, a critical neural transcription factor. Therefore, our study showed that the *Pou3f1-*miR-29b*-Dnmt3a* regulatory axis was active at the initial stage of neural differentiation and regulated the determination of cell fate.

## Highlights

• MiR-29b promoted NTE differentiation and inhibited NCC differentiation from ESCs
• MiR-29b targeted *Dnmt3a* to regulate neural differentiation
• MiR-29b mediated the function of *Pou3f1*
• The *Pou3f1*-miR-29b-*Dnmt3a* axis regulated the cell fate determination

## Graphical Abstract

**Figure:**
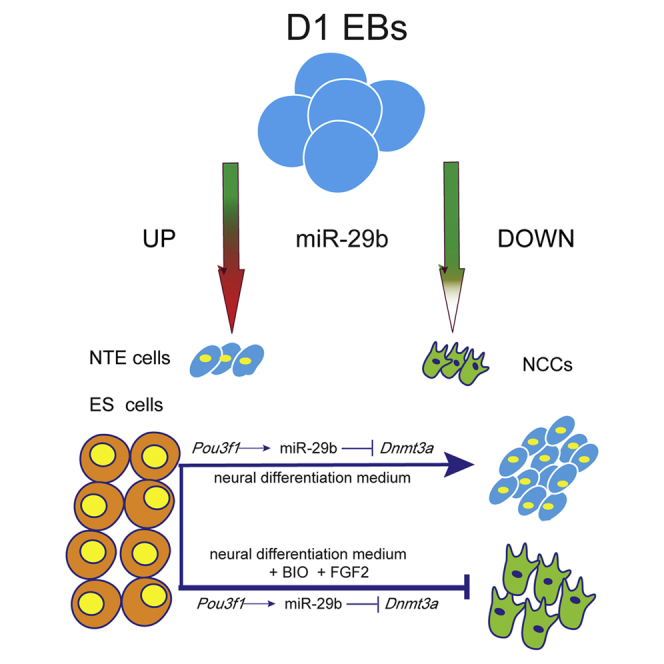

## Teaser

In this article, Kang and colleagues demonstrated that miR-29b mediated the function of a critical neural transcription factor *Pou3f1* to promote the differentiation of ESCs into NTE cells and inhibit their differentiation into NCCs by targeting *Dnmt3a*. This study showed that the *Pou3f1*-miR-29b-*Dnmt3a* regulatory axis was active at the initial stage of neural differentiation and regulated cell fate determination.

## Introduction

The structure and function of the nervous system are highly complex, and its development and differentiation have been extensively studied. During the gastrula stage, the entire nervous system is derived from a layer of epithelial-like cells on the dorsal surface, which is the neural plate formed by the ectoderm (Meulemans and Bronner-Fraser, 2004). During the neural development stage, both sides of the neural fold rise and gradually fuse from the front to the back. During this process of neural plate forming the neural tube, a group of neuroepithelial cells at the edge of both sides of the neural plate are separated from the dorsal wall of the neural tube to form a longitudinal strip of cells, which is clearly distinguished from the neural tube and epithelial cells that cover it; this strip is called the neural crest (Mayor and Theveneau, 2013). In subsequent developmental steps, the neural tube forms the entire CNS, whereas neural crest cells (NCCs) can differentiate into sensory neurons and Schwann cells in the peripheral nervous system and into a variety of specific cells, including smooth muscle cells and melanocytes (Bhatt et al., 2013). A single-cell lineage analysis showed that individual cells within the dorsal neural tube can contribute to both CNS- and neural crest-derived tissues, indicating that during the developmental process, the original neuroectodermal cells can differentiate into the neural tube and neural crest, which exhibit different properties (Bronner-Fraser and Fraser, 1988). However, the regulatory mechanism that controls the differentiation of neuroectodermal cells into the neural tube and neural crest during this developmental process remains unknown.

MicroRNAs (miRNAs) are a group of small RNA molecules that can specifically bind to the 3′ UTR or coding regions of target gene mRNA to negatively regulate target genes at the post-transcription level (Hobert, 2008). An increasing number of studies have shown that miRNAs play an important role in the regulation of cell differentiation during neural development (Fineberg et al., 2009, Shi et al., 2010). For example, miR-9 and miR-124 directly inhibit *Rest* and *Tlx* to promote the differentiation of neural stem cells into neurons (Conaco et al., 2006, Zhao et al., 2009). In the ventral spinal cord, miR-17-3p directly inhibits the transcription factor *Oligo2* to regulate the differentiation of motor neurons and V1 interneurons (Chen et al., 2011). Gessert et al. (2010) showed that the loss of miR-200, miR-96, and miR-196a resulted in differentiation restriction and inhibited the migration of NCCs in *Xenopus*. Moreover, miR-452, which is highly expressed in the neural crest, influences the migration and differentiation of NCCs by regulating epithelial-mesenchymal transition (Sheehy et al., 2010). Thus, it is interesting to study the role of miRNAs in controlling the differentiation of neuroectodermal cells into neural tube epithelial (NTE) cells and NCCs in a neural differentiation system.

Embryonic stem cells (ESCs) are derived from the inner cell mass, and can differentiate into cells of all tissue types in the body and exhibit unlimited self-renewal (Evans and Kaufman, 1981, Thomson et al., 1998). *In vitro*, ESCs can differentiate into NTE cells (Watanabe et al., 2005, Zhang, 2006) and NCCs (Lee et al., 2007, Liu et al., 2012, Minamino et al., 2015). Therefore, ESCs are an excellent model for the study of early development of the nervous system and the regulatory mechanisms that determine the differentiation of NTE cells and NCCs. Several genes that are critical for the neural differentiation of ESCs and miRNAs that play important roles in the determination of neural differentiation fate have been identified *in vitro*. Specifically, miR-302 and miR-371 family members must be inhibited during neural differentiation, and the overexpression of miR-371 family members blocks neural differentiation (Kim et al., 2011, Rosa and Brivanlou, 2010). Moreover, Du et al. (2013) showed that miR-200 and miR-96 inhibited the neural differentiation of human ESCs (hESCs) by targeting *ZEB* and *PAX6*, respectively.

In this study, we used the differentiation of ESCs into NTE cells and NCCs as a model to show that the expression of miR-29b was upregulated in NTE cells and downregulated in NCCs compared with the initial stage of differentiation. Furthermore, the overexpression of miR-29b promoted the differentiation of ESCs into NTE cells and inhibited differentiation into NCCs. A mechanistic study revealed that miR-29b targets *DNA methyltransferase 3a* (*Dnmt3a*) to regulate neural differentiation. Specifically, the overexpression of *Dnmt3a* during differentiation offset the ability of miR-29b to promote NTE cell differentiation and to inhibit NCC differentiation. In addition, miR-29b also mediated the function of *Pou3f1*, a critical transcription factor in neural differentiation. Specifically, POU3f1 binds upstream of miR-29b to promote the expression of miR-29b, and the inhibition of miR-29b offsets the ability of *Pou3f1* overexpression to promote the differentiation of ESCs into NTE cells.

## Results

### MiR-29b Exhibits a Discriminating Expression Level between NTE Cells and NCCs

**Figure 1:**
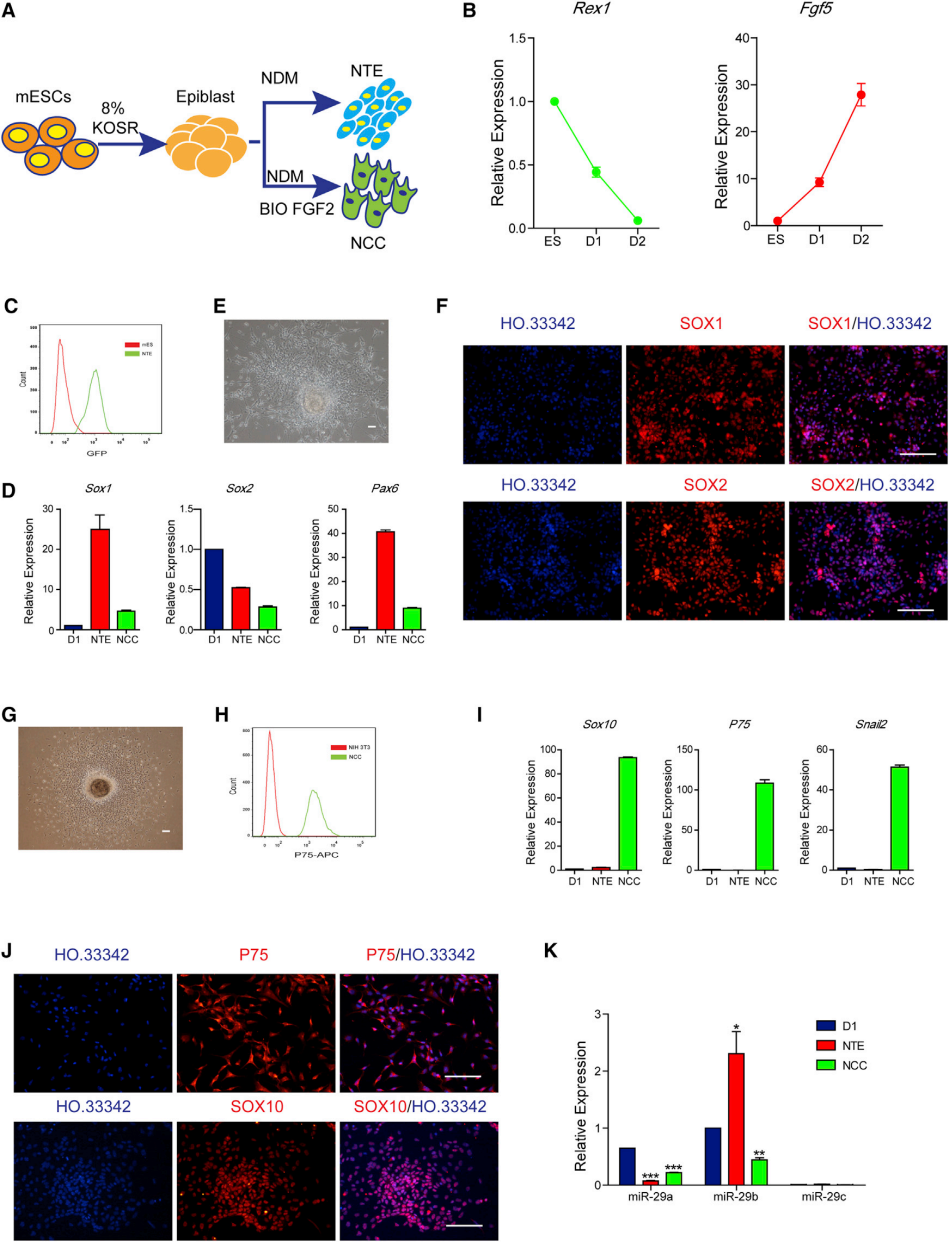
MiR-29b Exhibits a Discriminating Expression Level between NTE Cells and NCCs (A) Schematic showing the procedure for mESC differentiation into NTE and NCC. (B) The expression level of *Rex1* was downregulated and that of *Fgf5* was upregulated as verified by qPCR during the differentiation from embryonic stem cell (ES) to D2. (C) FACS analyzed the positive ratio of SOX1-GFP of mESC-NTE cells (green line) and undifferentiated ESCs (red line). (D) The neural lineage-associated genes *Sox1*, *Sox2*, and *Pax6* were upregulated as verified by qPCR in NTE cells. (E) The epithelial cells were observed after NTE EBs had attached to a Matrigel-coated surface. (F) Immunofluorescence assays of SOX1 and SOX2 in NTE cells. (G) The mesenchymal-like cells were observed to migrate out of the spheres after NCC EBs had attached to a Matrigel-coated surface. (H) FACS analyzed the positive ratio of P75 of mESC-NCCs (green line) and NIH-3T3 (red line). (I) The neural crest-associated genes *Sox10*, *P75*, and *Snail2* were upregulated as verified by qPCR in NCCs. (J) Immunofluorescence assays of P75 and SOX10 in NCCs. (K) qPCR measured the expression levels of miR-29 family of the NTE cells, NCCs, and D1 EBs. Means ± SEM from n = 3 independent experiments. ^∗^p < 0.05, ^∗∗^p < 0.01, ^∗∗∗^p < 0.001 versus the control. Scale bars, 100 μm.

To study the regulatory mechanism that determines cell fate at the early stage of neural differentiation of ESCs, we used the 46c cell line to establish NTE and NCC differentiation systems (Figure 1A). After differentiation for 2 days, the ESCs formed embryoid bodies (EBs); in addition, the expression of the pluripotent gene *Rex1* decreased and the expression of the epiblast-specific gene *Fgf5* was upregulated (Figure 1B), indicating that the cells had already differentiated to epiblast status. After continuous differentiation in neural differentiation medium for 4 days, the EBs could then be differentiated into SOX1-GFP-positive cells. Flow cytometry revealed that the proportion of SOX1-GFP-positive cells reached 93.4% (Figure 1C), and qPCR showed that the *Sox1*, *Sox2*, and *Pax6* genes were upregulated compared with the initial stage of differentiation (day 1 [D1] EBs) (Figure 1D). After the EBs had attached to Matrigel-coated culture dishes, epithelial cells were observed (Figure 1E). Immunofluorescence staining also showed SOX1- and SOX2-positive cells (Figure 1F). These results showed that ESCs differentiated into NTE cells. On D2 of differentiation, EBs were cultured in neural differentiation medium containing a glycogen synthase kinase 3β inhibitor (BIO) and fibroblast growth factor 2 (FGF2) and were allowed to continuously differentiate for 4–6 days. After EBs attached to Matrigel-coated culture dishes, many mesenchymal-like cells were observed migrating out of the spheres (Figure 1G); these cells preferentially adhere to and proliferate on a Matrigel-coated surface, and flow cytometry revealed that these cells were positive for P75 (Figure 1H). qPCR revealed that these cells expressed high levels of the genes *Sox10*, *P75*, and *Snail2* (Figure 1I), and immunofluorescence staining also showed P75- and SOX10-positive cells (Figure 1J), indicating they were NCCs. NTE cells and NCCs expressed miR-29 family members. Specifically, compared with the D1 EBs, miR-29b expression was upregulated in NTE cells and downregulated in NCCs, whereas miR-29a was downregulated in both NTE cells and NCCs, while miR-29c expression was not detected (Figure 1K). The differential expression of miR-29b in NTE cells and NCCs suggested that it might be involved in regulating the differentiation fates of these two types of cells.

### MiR-29b Is Required for NTE Differentiation

**Figure 2:**
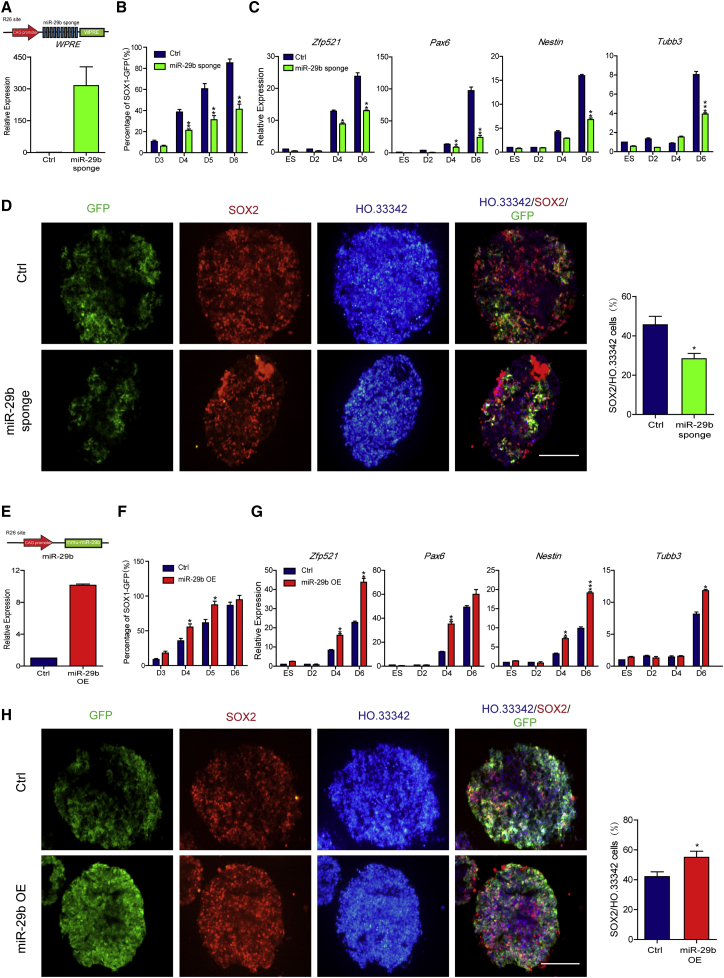
MiR-29b Is Required for NTE Differentiation (A) Diagram of insertion of a CAG promoter driving 10-copy miR-29b sponge into ROSA26 site and the expression level of miR-29b sponge verified by qPCR. (B–D) Inhibiting miR-29b reduced the efficiency of NTE differentiation, as shown by FACS (B) and qPCR (C) analysis during the NTE differentiation period, and immunofluorescence assays on D5 as well as the quantification of SOX2-positive cells (D). (E) Diagram of insertion of a CAG promoter driving miR-29b cassette into ROSA26 site and the overexpression efficiency of miR-29b verified by qPCR. (F–H) Overexpressing miR-29b improved the efficiency of NTE differentiation, as shown by FACS (F) and qPCR (G) analysis during the NTE differentiation period, and immunofluorescence assays on D5 as well as the quantification of SOX2-positive cells (H). Means ± SEM from n = 3 independent experiments. ^∗^p < 0.05, ^∗∗^p < 0.01, ^∗∗∗^p < 0.001 versus the control. Scale bars, 100 μm.

To study the effect of miR-29b on the differentiation of ESCs into NTE cells, we used the miRNA sponge strategy, which contains multiple tandem binding sites for a miRNA of interest to compete with target genes for interacting with miRNA (Ebert et al., 2007). Using the site-directed integration method, we established an miR-29b inhibiting cell line by inserting a CAG promoter driving ten copies of sponge sequence fused with a *WPRE* sequence into the ROSA26 site. The miR-29b sponge expressed significantly higher level as detected by qPCR for the *WPRE* sequence (Figure 2A). The expression of the miR-29b sponge showed no marked influence on the pluripotency maintenance of ESCs, as comparable expression levels of pluripotent marker genes, such as *Oct4*, *Nanog*, and *Sox2*, were detected in the miR-29b sponge overexpressing ESC line via qPCR (Figure S1A). During NTE differentiation, the proportion of SOX1-GFP-positive cells in the miR-29b sponge group significantly decreased (Figure 2B), and the expression levels of the NTE marker genes *Zfp521*, *Pax6*, *Nestin*, and *Tubb3* significantly decreased (Figure 2C). In addition, immunofluorescence staining showed that the proportions of SOX1-GFP-positive and SOX2-positive cells in the miR-29b sponge group decreased on D5 of differentiation (Figure 2D).

Furthermore, we stably expressed miR-29b in ESCs. A DNA fragment containing the miR-29b sequence driven by the CAG promoter was inserted into the ROSA26 site. The miR-29b overexpressed (OE) ESCs expressed an 8- to 10-fold higher level of miR-29b than control cells as detected by qPCR (Figure 2E). In addition, miR-29b overexpression did not affect the pluripotency maintenance of ESCs, as comparable expression levels of pluripotent marker genes, such as *Oct4*, *Nanog*, and *Sox2*, were detected in the miR-29b OE cell line via qPCR (Figure S1B). During NTE differentiation, the proportion of SOX1-GFP-positive cells in the miR-29b OE group significantly increased (Figure 2F), and the expression levels of the NTE marker genes *Zfp521*, *Pax6*, *Nestin*, and *Tubb3* significantly increased (Figure 2G). In addition, immunofluorescence staining showed that the proportions of SOX1-GFP-positive and SOX2-positive cells in the miR-29b OE group increased on D5 of differentiation (Figure 2H). These results indicated that miR-29b is necessary for NTE differentiation and that the overexpression of miR-29b promotes NTE differentiation.

### MiR-29b Inhibits NCC Differentiation

**Figure 3:**
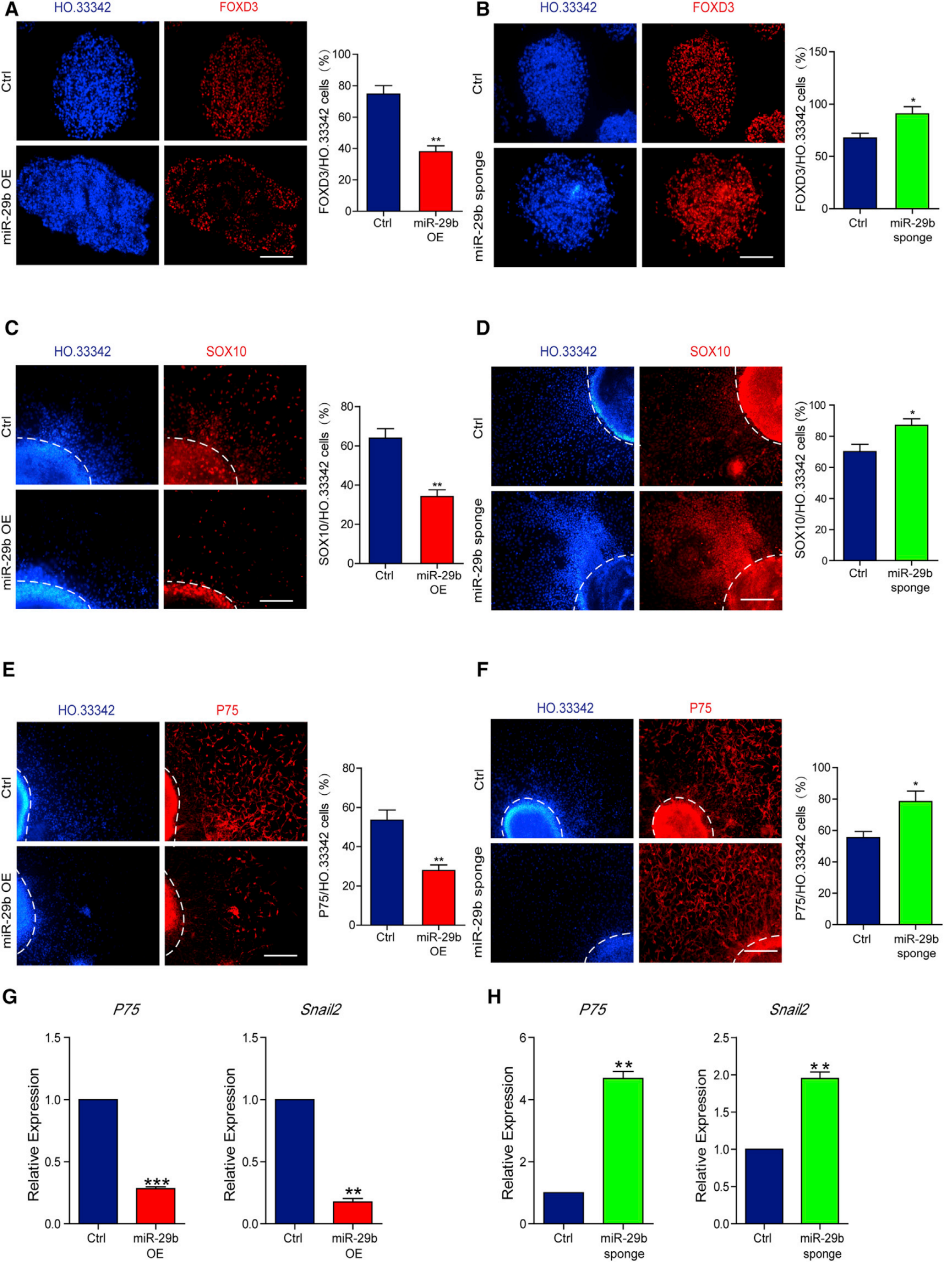
MiR-29b Inhibits NCC Differentiation (A and B) Immunofluorescence assays of FOXD3 on D4 showed that overexpressing miR-29b reduced FOXD3-positive cells (A) and inhibiting miR-29b increased FOXD3-positive cells (B). Quantification of FOXD3-positive cells is shown on the right. (C and D) Immunofluorescence assays of SOX10 on D10 showed that overexpressing miR-29b reduced SOX10-positive cells (C) and inhibiting miR-29b increased SOX10-positive cells (D). The dashed lines indicate the region of the sphere. Quantification of SOX10-positive cells is shown on the right. (E and F) Immunofluorescence assays of P75 on D12 showed that overexpressing miR-29b reduced P75-positive cells (E) and inhibiting miR-29b increased P75-positive cells (F). The dashed lines indicate the region of the sphere. Quantification of P75-positive cells is shown on the right. (G and H) qPCR measured the mRNA levels of NCC markers *P75* and *Snail2* on D12 of NCC differentiation cells, and showed that overexpressing miR-29b decreased the expression levels of neural crest-related genes (G) and inhibiting miR-29b increased the expression levels of neural crest-related genes (H). Means ± SEM from n = 3 independent experiments. ^∗^p < 0.05, ^∗∗^p < 0.01, ^∗∗∗^p < 0.001 versus the control. Scale bars, 100 μm.

We also studied the ability of miR-29b to regulate the differentiation of ESCs into NCCs. On D4 of differentiation, we measured the expression of the early NCC transcription factor *FoxD3* in EB sections. Compared with the control group, the miR-29b OE group exhibited a significant decrease in the number of FOXD3-positive cells (Figure 3A), whereas the miR-29b sponge group showed an increase in the number of FOXD3-positive cells (Figure 3B). After the cells attached to Matrigel-coated culture dishes, many mesenchymal-like cells were observed migrating out from the EBs in the miR-29b sponge group, whereas the migrating cells in the miR-29b OE group were primarily neuronal-like cells. On D10, we measured the expression of the NCC-specific transcription factor *Sox10* in migrating cells. We found that the number of SOX10-positive cells in the miR-29b OE group significantly decreased (Figure 3C) and that the number of SOX10-positive cells in the miR-29b sponge group increased (Figure 3D). On D12, we measured the expression of the mature NCC marker gene *P75* and found that the number of P75-positive cells in the miR-29b OE group significantly decreased (Figure 3E), whereas the number of P75-positive cells in the miR-29b sponge group increased (Figure 3F). The decreased expression levels of *P75* and *Snail2* in miR-29b OE cells were consistent with the immunofluorescence results (Figure 3G). In addition, we found significantly increased expression levels of *P75* and *Snail2* in the miR-29b sponge group (Figure 3H). Therefore, we concluded that miR-29b inhibits the differentiation of ESCs into NCCs and that the inhibition of miR-29b by the sponge strategy promoted the differentiation of NCCs.

### MiR-29b Affects Fate Determination between NTE Cells and NCCs by Targeting *Dnmt3a*

**Figure 4:**
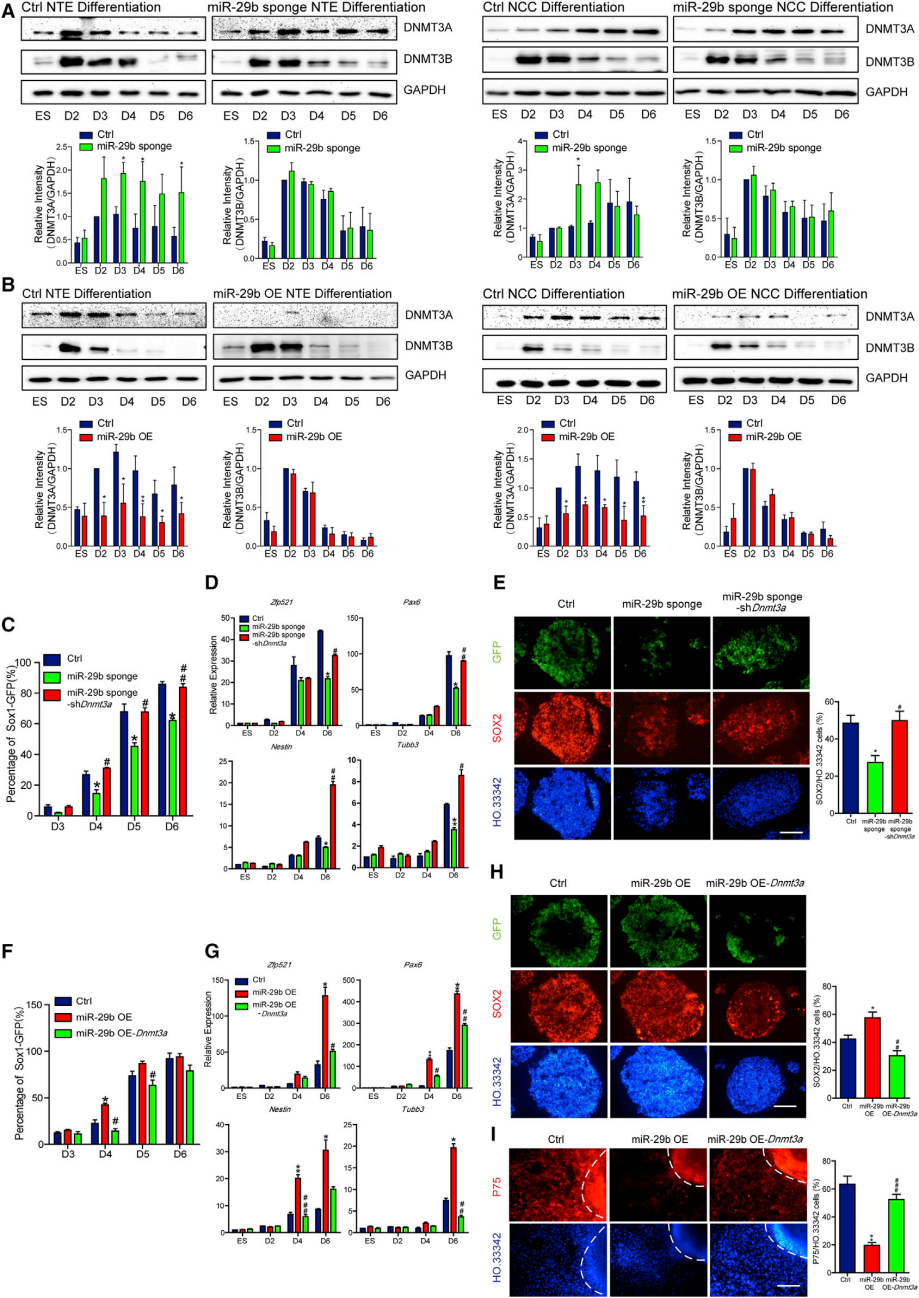
MiR-29b Affects Fate Determination between NTE Cells and NCCs by Targeting *Dnmt3a* (A and B) Western blotting analyzed the protein levels of DNMT3A and DNMT3B in control (Ctrl) and miR-29b sponge cell line (A) or Ctrl and miR-29b OE cell line (B) during NTE and NCC differentiation. GAPDH is the normalization control. The protein abundance of DNMT3A and DNMT3B was quantified with normalization by signals of GAPDH. (C–E) Knocking down *Dnmt3a* rescued the defection of NTE differentiation by inhibiting miR-29b, as shown by FACS (C) and qPCR (D) during NTE differentiation, and immunofluorescence on D5 of NTE differentiation EBs as well as the quantification of SOX2-positive cells (E). (F–H) Overexpressing *Dnmt3a* offset the promotion of NTE differentiation by miR-29b, as shown by FACS (F) and qPCR (G) during NTE differentiation, and immunofluorescence on D5 of NTE differentiation EBs as well as the quantification of SOX2-positive cells (H). (I) Immunofluorescence assays of P75 on D12 of NCC differentiation cells showed that overexpressing *Dnmt3a* rescued the defection of NCC differentiation by miR-29b. The dashed lines indicate the region of the sphere. Means ± SEM from n = 3 independent experiments. ^∗^p < 0.05, ^∗∗^p < 0.01 versus the control; ^#^p < 0.05, ^##^p < 0.01, ^###^p < 0.001 versus the miR-29b sponge group (C–E) or miR-29b OE group (F–I). Scale bars, 100 μm.

*Dnmt3a* and *Dnmt3b* are considered the most important miR-29b target genes (Cicchini et al., 2015, Guo et al., 2013, Meunier et al., 2012). Therefore, we first detected the expression levels of *Dnmt3a* and *Dnmt3b* during the differentiation of ESCs into NTE cells and NCCs. The western blotting results showed that the protein level of DNMT3A increased on D2 of differentiation and subsequently decreased during NTE differentiation, whereas DNMT3A was continuously expressed during NCC differentiation (Figure S2A). The protein level of DNMT3B increased on D2 of NTE and NCC differentiation and then subsequently decreased (Figure S2A). To assess whether the effect of miR-29b was mediated by *Dnmt3a* and *Dnmt3b*, the protein levels of these DNMTs were measured after the inhibition and overexpression of miR-29b during NTE and NCC differentiation. Compared with the control group, the miR-29b sponge group exhibited a significant increase in DNMT3A expression and no change in DNMT3B expression (Figure 4A); conversely, DNMT3A expression decreased, and DNMT3B expression remained unchanged in miR-29b OE cells (Figure 4B). Therefore, we speculated that miR-29b may target *Dnmt3a* to regulate differentiation.

As miR-29b has been reported to regulate the Wnt/β-catenin signaling pathway (Kapinas et al., 2010, Shin et al., 2014, Subramanian et al., 2014), which is important for neural crest differentiation (Sauka-Spengler and Bronner-Fraser, 2008) and neurogenesis (Shin et al., 2014), we further investigated the effect of miR-29b on the Wnt/β-catenin signaling pathway during NTE and NCC differentiation. The regulation of Wnt/β-catenin signaling by miR-29b was evaluated by using a TOPFlash/FOPFlash reporter system (Veeman et al., 2003) and by detecting the expression levels of total and active β-catenin protein (CTNNB1). TOPFlash activity was increased in NCC differentiation compared with NTE differentiation, and neither overexpression nor inhibition of miR-29b affected TOPFlash activity (Figure S2B). The western blotting results showed that total and active CTNNB1 expression levels were not affected by overexpression or inhibition of miR-29b during NCC differentiation (Figures S2C and S2D). These results indicated that miR-29b may not regulate the Wnt/β-catenin signaling pathway during the early stage of neural differentiation.

To confirm that miR-29b targets *Dnmt3a* to regulate differentiation, we infected cells in the miR-29b sponge group with plko-sh*Dnmt3a* lentiviruses to establish a miR-29b sponge-sh*Dnmt3a* cell line. Knockdown was validated by measuring the protein level of DNMT3A via western blotting (Figure S2E). During differentiation into NTE cells, *Dnmt3a* knockdown offset the inhibition of NTE differentiation after the suppression of miR-29b. The number of SOX1-GFP-positive cells in the miR-29b sponge-sh*Dnmt3a* group significantly increased compared with those in the miR-29b sponge group (Figure 4C). Furthermore, the expression levels of the neural differentiation-related genes *Zfp521*, *Pax6*, *Nestin*, and *Tubb3* in the miR-29b sponge-sh*Dnmt3a* group significantly increased compared with those in the miR-29b sponge group (Figure 4D). Immunofluorescence staining on D5 of differentiation also confirmed that the proportions of SOX1-GFP-positive and SOX2-positive cells significantly increased in the miR-29b sponge-sh*Dnmt3a* group (Figure 4E). These results indicated that *Dnmt3a* knockdown offset the inhibition of NTE differentiation following the suppression of miR-29b.

Furthermore, we established an miR-29b OE-*Dnmt3a* cell line that overexpressed *Dnmt3a* in the miR-29b OE group. The overexpression of DNMT3A was validated via western blotting (Figure S2F). During differentiation into NTE cells, *Dnmt3a* overexpression offset the promotion of NTE differentiation by miR-29b. The number of SOX1-GFP-positive cells in the miR-29b OE-*Dnmt3a* group significantly decreased compared with that in the miR-29b OE group (Figure 4F). Furthermore, the expression levels of the neural differentiation-related genes *Zfp521*, *Pax6*, *Nestin*, and *Tubb3* in the miR-29b OE-*Dnmt3a* group significantly decreased compared with those in the miR-29b OE group (Figure 4G). Immunofluorescence staining on D5 of differentiation also confirmed that the proportions of SOX1-GFP-positive and SOX2-positive cells significantly decreased in the miR-29b OE-*Dnmt3a* group compared with the miR-29b OE group (Figure 4H). Moreover, *Dnmt3a* overexpression offset the inhibition of NCC differentiation by miR-29b. Immunofluorescence staining on D12 of differentiation showed that compared with the miR-29b OE group, the miR-29b OE-*Dmnt3a* group exhibited a significant increase in the number of P75-positive cells (Figure 4I). These results indicated that miR-29b regulates the fate of ESC differentiation into NTE cells and NCCs by targeting *Dnmt3a*.

### MiR-29b Acts Downstream of *Pou3f1* to Drive Mouse ESCs into the NTE Lineage

**Figure 5:**
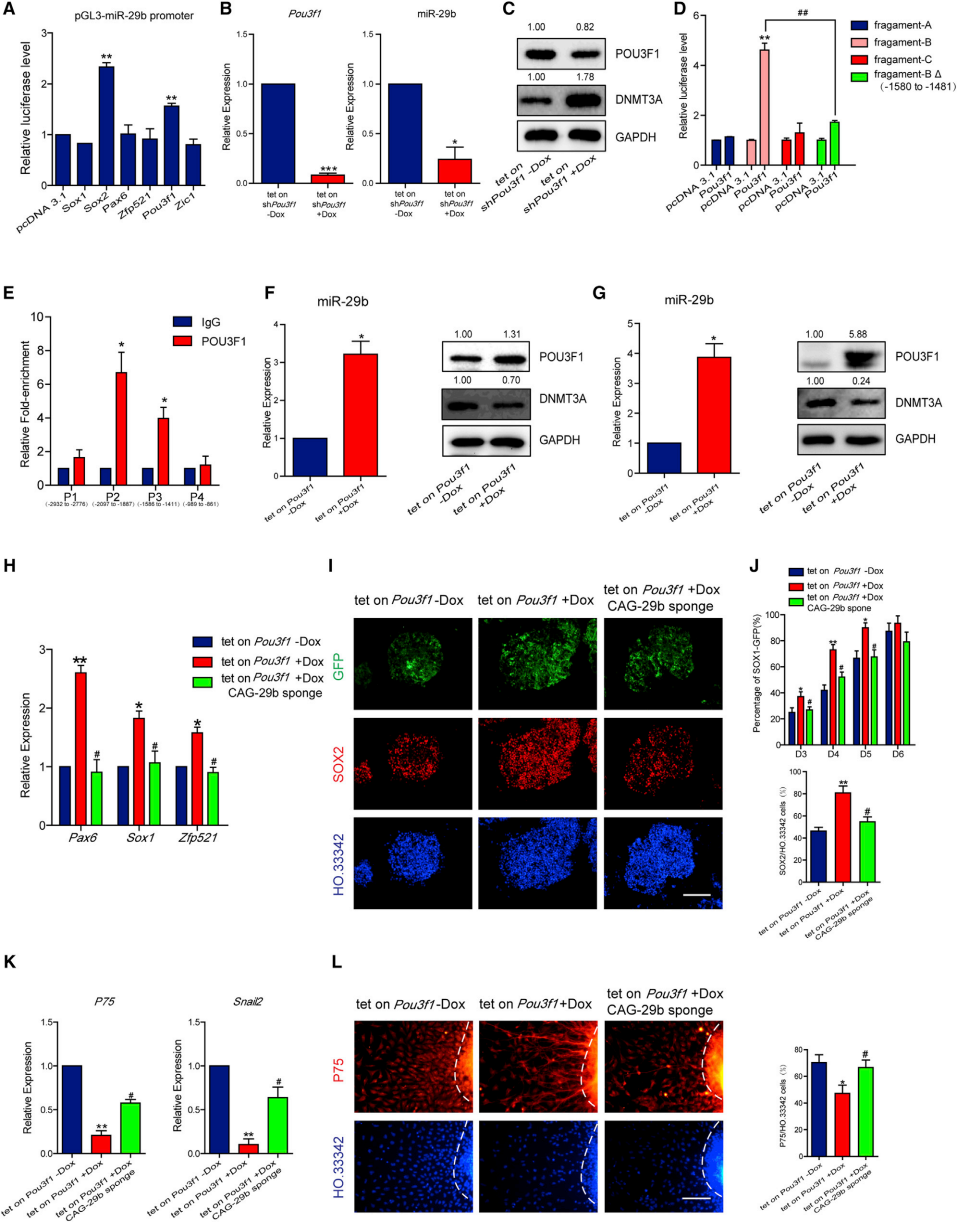
MiR-29b Acts Downstream of *Pou3f1* to Drive Mouse ESCs into the NTE Lineage (A) Luciferase reporter assay in 3T3 cells transfected control (pcDNA3.1), *Sox1*, *Sox2*, *Pax6*, *Zfp521*, *Pou3f1*, and *Zic1*, respectively, with pGL3-miR-29b promoter. (B and C) qPCR (B) and western blotting (C) in tet-on sh*Pou3f1* cell line with and without Dox showed that *Pou3f1* knockdown reduced the expression of miR-29b and increased the protein level of DNMT3A. The protein abundance of POU3F1 and DNMT3A was quantified with normalization by signals of GAPDH. (D) Luciferase reporter assay in 3T3 cells transfected control (pcDNA3.1) and *Pou3f1*, respectively, with pGL3 fragments of miR-29b promoter. (E) ChIP assay showed enrichment of POU3F1 at the promoter region of miR-29b. (F and G) Western blotting showed increased level of POU3F1 and decreased level of DNMT3A in tet-on *Pou3f1* cell line with and without Dox on D3 of NTE (F) and NCC (G) differentiation. The protein abundance of POU3F1 and DNMT3A was quantified with normalization by signals of GAPDH. Overexpression of *Pou3f1* upregulates the expression level of miR-29b on D3 of NTE (F) and NCC (G) differentiation, as verified by qPCR. (H–J) Inhibiting miR-29b offset the promotion of NTE differentiation by *Pou3f1*, as shown by qPCR (H), FACS (J), and immunofluorescence (I) as well as the quantification of SOX2-positive cells on D5 of NTE differentiation EBs. (K and L) Inhibiting miR-29b rescue the defection of NCC differentiation by *Pou3f1*, as shown by qPCR (K) and immunofluorescence as well as quantification of P75-positive cells (L) on D12 of NCC differentiation. The dashed lines indicate the region of the sphere. Means ± SEM of n = 3 independent experiments. ^∗^p < 0.05, ^∗∗^p < 0.01, ^∗∗∗^p < 0.001 versus the control; ^#^p < 0.05, ^##^p < 0.01 versus the fragment-B group (D) or tet-on Pou3f1 + Dox group (H–L). Scale bars, 100 μm.

To identify upstream transcription factors that regulate miR-29b, we used a pGL3 luciferase plasmid containing the 3-kb region upstream of the miR-29b to identify transcription factors that are upregulated in NTE cells and activate miR-29b. The results showed that *Pou3f1* and *Sox2* enhanced the luciferase activity (Figure 5A). Chromatin immunoprecipitation (ChIP) experimental results indicated that POU3F1 bound at the upstream region of miR-29b, whereas the binding of SOX2 was not detected (Figure S3A). Furthermore, we established ESC lines in which either *Pou3f1* or *Sox2* was knocked down. After 2 days of differentiation, *Sox2* knockdown did not affect miR-29b expression (Figure S3B), whereas *Pou3f1* knockdown reduced miR-29b expression (Figure 5B) and increased the protein level of DNMT3A (Figure 5C). We also found the transcriptional activity of *Pou3f1* depended on its POU domain, as *Pou3f1ΔPOU* lost the ability to stimulate luciferase expression (Figure S3C).

To further examine the potential site that was essential for POU3F1-activated transcription, we used MAST (Motif Alignment and Search Tool) (Bailey et al., 2009) to analyze the miR-29b upstream region with the POU3F1 DNA-binding motif (Figure S3D) from the UniPROBE database (Hume et al., 2015). A potential POU3F1-binding site has been identified, located in the upstream of miR-29b (−1,539 to −1,518 bp region) (Figure S3E). Based on this analysis, we divided the miR-29b upstream region into three fragments, including fragment A (−3,000 to −2,001 bp), fragment B (−2,000 to −1,001 bp), and fragment C (−1,000 to −1 bp). We then inserted these fragments into pGL3 vectors and performed co-transfection with *Pou3f1* for a luciferase assay. The results showed that only fragment B containing this predicted POU3F1-binding site conferred peak transcriptional activity (Figure 5D), whereas fragments A and C had negligible effects. Deletion of a 100-bp region (−1,580 to −1,481) around this site in fragment B negated the activation effect of POU3F1 (Figure 5D). ChIP experimental results showed that in the upstream region of the miR-29b, only the region that contained predicated POU3F1-binding site was enriched in POU3F1 immunoprecipitates, whereas the binding of POU3F1 was not detected in the nearby regions (Figures 5E and S3F). The same region was also enriched for the promoter-specific histone modification H3K4me3 and H3K9Ac (Agalioti et al., 2002, Guenther et al., 2007, Seila et al., 2008, Wang et al., 2008), indicating that this region was the promoter of miR-29b and that POU3F1 directly bound to the promoter of miR-29b (Figure S3G). Therefore, we hypothesized that miR-29b is regulated by *Pou3f1* during neural differentiation.

To further study the effect of *Pou3f1* on miR-29b-mediated regulation of neural differentiation, we established an ESC line in which *Pou3f1* or *Pou3f1ΔPOU* expression could be induced by doxycycline (Dox) (Figure S3H). During differentiation, induced *Pou3f1* expression activated miR-29b expression and accordingly decreased the protein level of DNMT3A in both NTE (Figure 5F) and NCC (Figure 5G) differentiation, whereas *Pou3f1ΔPOU* had no effect on the activation of miR-29b expression (Figure S3I). Induced *Pou3f1* expression promoted NTE differentiation and increased *Pax6*, *Sox1*, and *Zfp521* expression levels (Figure 5H), while overexpression of *Pou3f1ΔPOU* did not show this effect (Figure S3J). Accordingly, the inhibition of miR-29b via the overexpression of the miR-29b sponge offset the promotion of NTE differentiation by *Pou3f1* overexpression. Specifically, qPCR results showed that *Pax6*, *Sox1*, and *Zfp521* expression levels decreased (Figure 5H), the immunofluorescence staining results also showed that the proportions of SOX1-GFP-positive and SOX2-positive cells decreased on D5 of differentiation in the tet-on *Pou3f1* + Dox CAG-29b sponge group compared with the tet-on *Pou3f1* + Dox group (Figure 5I). The number of SOX1-GFP-positive cells in the tet-on *Pou3f1* + Dox CAG-29b sponge group was significantly decreased compared with that in the tet-on *Pou3f1* + Dox group (Figure 5J). During differentiation into NCCs, Dox-induced expression of *Pou3f1* decreased the expression levels of both *P75* and *Snail2* (Figure 5K). The immunofluorescence results showed that the number of migrating P75-positive NCCs significantly decreased, indicating that *Pou3f1* inhibited NCC differentiation (Figure 5L). Therefore, the expression levels of both *P75* and *Snail2* significantly increased when miR-29b was suppressed by the overexpression of the miR-29b sponge (Figure 5K). Immunofluorescence staining also showed that the number of P75-positive cells recovered (Figure 5L). Our results indicated that *Pou3f1* activates miR-29b and regulates neural differentiation via miR-29b. The ability of *Pou3f1* to promote NTE differentiation was offset by the inhibition of miR-29b, whereas the inhibition of miR-29b also offset the inhibition of NCC differentiation by *Pou3f1*.

### MiR-29b Regulates NTE versus NCC Fate Decision in hESC Neural Differentiation

**Figure 6:**
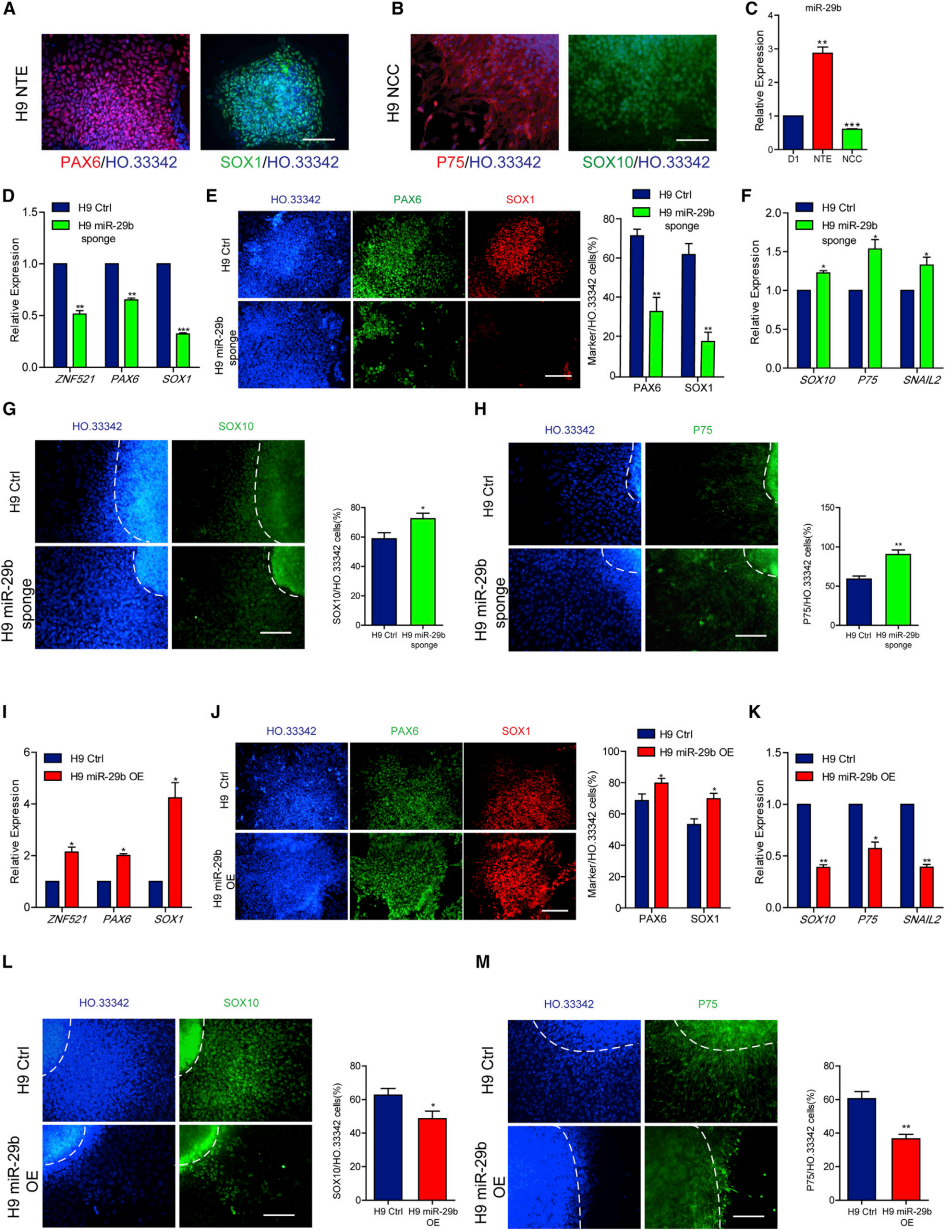
MiR-29b Regulates NTE versus NCC Fate Decision in Human ESC Neural Differentiation (A) Immunofluorescence assays of PAX6 and SOX1 in hESC-derived NTE cells. (B) Immunofluorescence assays of P75 and SOX10 in hESC-derived NCCs. (C) Compared with initiation differentiation cells (D1), miR-29b was upregulated in NTE cells and downregulated in NCCs as verified by qPCR. (D and E) Inhibiting miR-29b reduced the NTE differentiation efficiency of hESCs as shown by qPCR (D) and immunofluorescence of NTE differentiation as well as the quantification of PAX6- and SOX1-positive cells (E) on D12. (F–H) Inhibiting miR-29b improved the NCC differentiation efficiency of hESCs as shown by qPCR (F) and immunofluorescence (G and H) of NCC differentiation cells, and quantification of SOX10- and P75-positive cells, on D12. The dashed lines indicate the region of the sphere. (I and J) Overexpressing miR-29b improved the NTE differentiation efficiency of hESCs as shown by qPCR (I) and immunofluorescence (J) of NTE differentiation cells, and quantification of PAX6- and SOX1-positive cells, on D12. (K–M) Overexpressing miR-29b reduced the NCC differentiation efficiency of hESCs as shown by qPCR (K), and immunofluorescence (L and M) of NCC differentiation cells, and quantification of SOX10- and P75-positive cells, on D12. The dashed lines indicate the region of the sphere. Means ± SEM from n = 3 independent experiments. ^∗^p < 0.05, ^∗∗^p < 0.01, ^∗∗∗^p < 0.001 versus the control. Scale bars, 100 μm.

After showing that miR-29b regulates cell fate at the early stage of neural differentiation in mouse ESCs (mESCs), we attempted to recapitulate this effect in hESCs. Based on a reported method (Zhang et al., 2001, Liu et al., 2012), we differentiated hESCs into NTE cells and NCCs. The hESCs were maintained on mouse embryonic fibroblast (MEF) feeders and exhibited an undifferentiated phenotype. The hESCs were detached from MEFs and formed EBs to initiate the differentiation process (Figure S4A). For NTE differentiation, the EBs adhered to the culture surface from D7 and formed a rosette-like structure (Figure S4B); these columnar epithelial cells expressed PAX6 and SOX1 and were NTE cells (Figure 6A). For NCC differentiation, FGF2 and BIO were added to the culture medium at D4–D10. After the EBs were attached to the Matrigel-coated dishes, cells with a stellate morphology migrated out of the spheres (Figure S4C). These cells expressed P75 and SOX10 and were therefore NCCs (Figure 6B). In addition, miR-29b expression was upregulated in NTE cells and downregulated in NCCs compared with that in D1 EBs (Figure 6C). To study the regulatory function of miR-29b during the neural differentiation of hESCs, we used a site-directed integration method to insert a CAG promoter driving ten copies of sponge sequences fused with *WPRE* sequences into the AAVS1 site, and established an H9 miR-29b inhibiting cell line. The expression level of the *WPRE* in the miR-29b sponge was detected by qPCR (Figure S4D), and inhibition of miR-29b had no influence on the pluripotency maintenance of hESCs (Figure S4E). We also found that DNMT3A expression level increased in H9 miR-29b sponge cells (Figure S4F). On D12 of NTE differentiation, the expression levels of the NTE marker genes *ZNF521*, *PAX6*, and *SOX1* in the H9 miR-29b sponge group were significantly lower than the levels of the control group (Figure 6D). In addition, immunofluorescence staining showed that the proportions of PAX6 and SOX1 in the H9 miR-29b sponge group decreased (Figure 6E). In addition, on D12 of NCC differentiation, neural crest-related gene expression was assessed using qPCR, revealing that *SOX10*, *P75*, and *SNAIL2* expression levels were increased compared with the levels in control cells (Figure 6F). Moreover, immunofluorescence staining also showed that the proportions of SOX10- and P75-positive cells increased in the H9 miR-29b sponge group (Figures 6G and 6H).

Furthermore, by inserting a DNA fragment containing the miR-29b sequence driven by the CAG promoter into the AAVS1 site, we stably expressed miR-29b in hESCs. In these cells miR-29b expression was upregulated, as measured by qPCR (Figure S4G), and overexpression of miR-29b did not affect hESC pluripotency (Figure S4H). DNMT3A expression was decreased, as detected by western blotting (Figure S4I). After 12 days of NTE differentiation, the expression levels of the NTE marker genes *ZNF521*, *PAX6*, and *SOX1* in the H9 miR-29b OE group significantly increased compared with those in the control group (Figure 6I). In addition, immunofluorescence staining showed that the proportions of PAX6 and SOX1 in the H9 miR-29b OE group increased (Figure 6J). Moreover, on D12 of NCC differentiation, qPCR was used to detect neural crest-related gene expression and revealed that *SOX10*, *P75*, and *SNAIL2* expression levels decreased compared with the levels in the control group (Figure 6K). Immunofluorescence staining also showed that the proportions of SOX10- and P75-positive cells decreased in the H9 miR-29b OE group (Figures 6L and 6M). These results indicated that miR-29b also promotes NTE cell differentiation and inhibits NCC differentiation during the neural differentiation of hESCs.

## Discussion

Previous studies on the determination of cell fate during the early stage of neural differentiation mainly focused on signaling pathways and transcription factors. FGF, BMP, and Wnt signaling pathway were shown to be very important in neural crest differentiation (Sauka-Spengler and Bronner-Fraser, 2008). Two groups of transcription factors, neural plate border specifier genes (*Msx1*, *Pax3*, *Pax7*, and *Zic1*) and NCC specifier genes (*SoxE*, *FoxD3*, *Snail1*, and *Snail2*), mediated the upstream signals and regulated the occurrence, development, migration, and differentiation of NCCs (Meulemans and Bronner-Fraser, 2004). However, little is known regarding the function of non-coding RNAs, a large group of RNAs, in this process. We differentiated ESCs into NTE cells and NCCs and showed that miR-29b is an important molecule in the determination of neural tube versus neural crest fate during neural differentiation.

MiR-29b has been shown to target many molecules and regulate signaling pathways (Cheng et al., 2013, Leone et al., 2012, Tu et al., 2015, Vitiello et al., 2016, Xu et al., 2014). Previous studies showed miR-29b could either activate or negatively regulate the canonical Wnt signaling pathway (Kapinas et al., 2010, Subramanian et al., 2014). Significant upregulation of miR-29b has been found in neural stem cells and promotes neurogenesis though regulation of the canonical Wnt signaling pathway (Shin et al., 2014). In our study, using a TOPFlash/FOPFlash reporter system and detecting the protein levels of total and active CTNNB1 by western blotting, we found that during NTE and NCC differentiation, neither overexpression nor inhibition miR-29b affected the activation of the Wnt/β-catenin signaling pathway, indicating that miR-29b acts in a context-dependent manner and does not regulate Wnt/β-catenin signaling during the early stage of neural differentiation.

*Dnmt3a* and *Dnmt3b* are the most commonly reported molecules targeted by miR-29b (Cicchini et al., 2015, Guo et al., 2013). Our studies showed that during neural differentiation, miR-29b inhibits *Dnmt3a* expression to promote NTE cell differentiation and inhibit NCC differentiation but does not significantly affect the expression of *Dnmt3b*. This result was consistent with the results of previous studies of neural differentiation and neural development. Specifically, Martins-Taylor et al. (2012) showed that *Dnmt3b* knockout during ESC differentiation accelerated NTE cell and NCC differentiation. *Dnmt3b* knockout in *Wnt1-cre* and *Sox10-cre* mice had little influence on the specification of NCCs and only slightly affected the migration of SOX10-positive cells (Jacques-Fricke et al., 2012). Unlike the dispensable role of *Dnmt3b* in neural crest differentiation, Hu et al. (2012) showed that the inhibition of *Dnmt3a* significantly affected neural crest development but did not affect neural tube development during the development of chick embryos. Therefore, *Dnmt3a* is considered a critical molecule in the regulation of neural tube versus neural crest fate. However, the mechanism responsible for regulating the expression of *Dnmt3a* during neural development remains poorly understood. MiR-29b, which is highly expressed in NTE cells, targets and inhibits *Dnmt3a* expression, thus ensuring the differentiation fate of the neural tube.

*Pou3f1*, an important transcription factor at the initial stage of neural development that is highly expressed in the early stage of the neuroectoderm, activated miR-29b during differentiation. Loss of *Pou3f1* during mESC differentiation caused cells to remain at the epiblast stage, whereas overexpression of *Pou3f1* promoted the neural differentiation of mESCs. Further studies indicated that POU3F1 could bind to the promoter regions of *Zfp521* and *Pax6* to activate the expression of *Zfp521* and *Pax6*, thus initiating neural differentiation (Zhu et al., 2014). Our study showed that *Pou3f1* also regulates neural differentiation by regulating miR-29b expression. The ChIP experimental results showed that POU3F1 directly bound at the promoter region of miR-29b; *Pou3f1* overexpression activated the expression of miR-29b, and *Pou3f1* knockdown significantly decreased the expression of miR-29b. Moreover, the overexpression of *Pou3f1* inhibited neural crest differentiation, and this function was offset by inhibition of miR-29b. Therefore, our results demonstrated that the *Pou3f1*-miR-29b-*Dnmt3a* regulatory axis was active at the initial stage of neural differentiation and regulated the differentiation fate of cells. Specifically, the high level of *Pou3f1* expression in NTE cells induced miR-29b to inhibit *Dnmt3a* and block neuroepithelial cells from differentiating into NCCs, thus ensuring NTE differentiation.

The upstream regions of miRNAs include binding sites for a variety of transcription factors, and various molecules regulate miRNAs in different cells and biological processes. Accordingly, miR-29b is also regulated by many types of signals and transcription factors. During somatic cell reprogramming, miR-29b is regulated by the pluripotent gene *Sox2*. *Sox2* also inhibits *Dnmt3a* via miR-29b to promote the formation of reprogrammed pluripotent stem cells (Guo et al., 2013). Our current study showed that during neural differentiation, *Sox2* expressed in NTE cells did not bind to the upstream region of miR-29b, indicating that miR-29b could be regulated by different transcription factors in different biological processes.

ESCs exhibit multidirectional differentiation and are an excellent model for *in vitro* developmental studies. Studying the neural differentiation of hESCs can help elucidate the developmental and regulatory mechanisms of the human nervous system (Bajpai et al., 2010, Zhang, 2006). During the differentiation of hESCs into NTE cells and NCCs, miR-29b regulates cell fate determination between NTE cells and NCCs by regulating *DNMT3a* expression. This result was consistent with the regulation of neural differentiation by miR-29b in mESCs, which revealed that the mechanism of miR-29b that determines cell fate at the early stage of neural differentiation is conserved.

This study confirmed that miR-29b regulates the differentiation fates of NTE cells and NCCs during the neural differentiation of ESCs and elucidated the synergistic function of the *Pou3f1*-miR-29b-*Dnmt3a* regulatory axis in the regulation of stem cell differentiation. These results also shed light on the mechanism that regulates neural tube/neural crest fate at the early stage of human neural development.

## Experimental Procedures

### Mouse ESC Culture and Differentiation

The mESC Sox1-GFP (46c) was cultured on a feeder layer. The NTE differentiation was performed according to published protocols (Wang et al., 2011) and the NCC differentiation was performed according to a published protocol (Minamino et al., 2015). For additional details, see Supplemental Experimental Procedures.

### hESC Culture and Differentiation

The culture and NTE differentiation of hESCs was performed according to published protocols (Zhang et al., 2001). The NCC differentiation of hESC was performed according to a published protocol (Liu et al., 2012). For additional details, see Supplemental Experimental Procedures.

### RNA Extraction, cDNA Synthesis, and qPCR Analysis

Total RNA was extracted using RNAiso Plus (TaKaRa). Reverse transcription was performed using a PrimeScript RT reagent kit (TaKaRa) and miRcute miRNA First-Strand cDNA Synthesis Kit (Tiangen). qPCR was performed using a SYBR Premix Ex Taq Kit (TaKaRa) and miRcute miRNA qPCR Detection Kit (Tiangen).

### Statistical Analyses

All statistical data are presented as the mean ± SEM of at least three independent experiments. Statistical significance was calculated according to unpaired two-tailed Student's t tests using GraphPad Prism software. p < 0.05 was considered statistically significant. Values are indicated on graphs as ^∗^p < 0.05, ^#^p < 0.05, ^∗∗^p < 0.01, ^##^p < 0.01, ^∗∗∗^p < 0.001, and ^###^p < 0.001.

## Author Contributions

J.X. and Y.W. designed the experiments, acquired the data, and performed the analysis. G.L. contributed to plasmid construction. L.M., G.Y., and K.F. provided materials and contributed to technical assistance. X.G., W.J., G.W., and P.L. discussed the project conception and design. J.X., Y.W., and J.K. wrote the manuscript.

## Supplemental Information

Document S1. Supplemental Experimental Procedures, Figures S1–S4, and Tables S1–S6

Document S2. Article plus Supplemental Information
