# Supplementary material for: Mir-29b Mediates the Neural Tube versus Neural Crest Fate Decision during Embryonic Stem Cell Neural Differentiation
Source: Stem Cell Reports. 2017 Jul 27;9(2):571–86. doi: 10.1016/j.stemcr.2017.06.017 (PMC5550033; doi:10.1016/j.stemcr.2017.06.017)
Supplement: Document S1. Supplemental Experimental Procedures, Figures S1–S4, and Tables S1–S6 [file mmc1.pdf]

Stem Cell Reports, Volume *9*

Supplemental Information

Mir-29b Mediates the Neural Tube versus Neural Crest Fate Decision during Embryonic Stem Cell Neural Differentiation

Jiajie Xi, Yukang Wu, Guoping Li, Li Ma, Ke Feng, Xudong Guo, Wenwen Jia, Guiying Wang, Guang Yang, Ping Li, and Jiuhong Kang

Figure S1

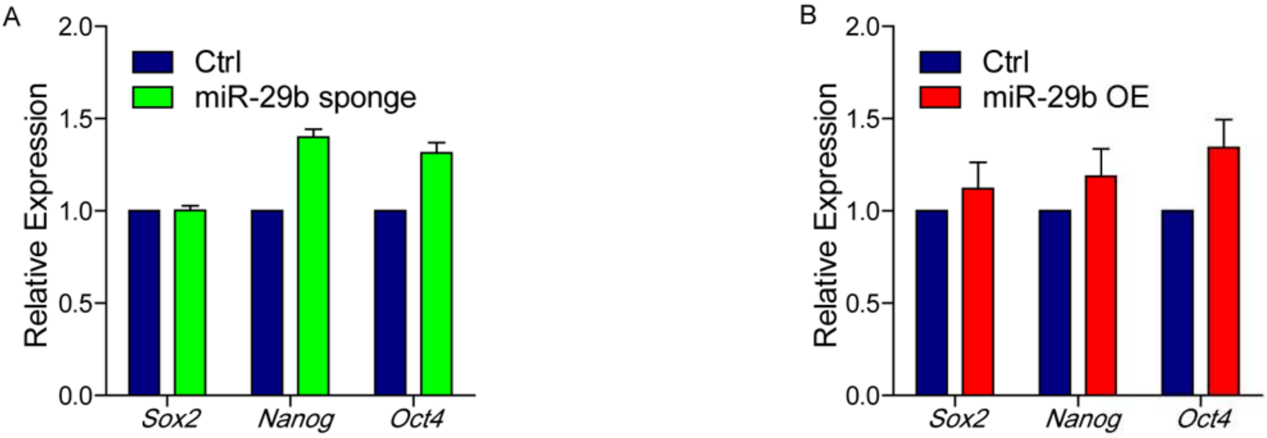
A
2.0
1.5
1.0
0.5
0.0
Relative Expression
Ctrl
miR-29b sponge
Sox2
Nanog
Oct4
B
2.0
1.5
1.0
0.5
0.0
Relative Expression
Ctrl
miR-29b OE
Sox2
Nanog
Oct4

Figure S2

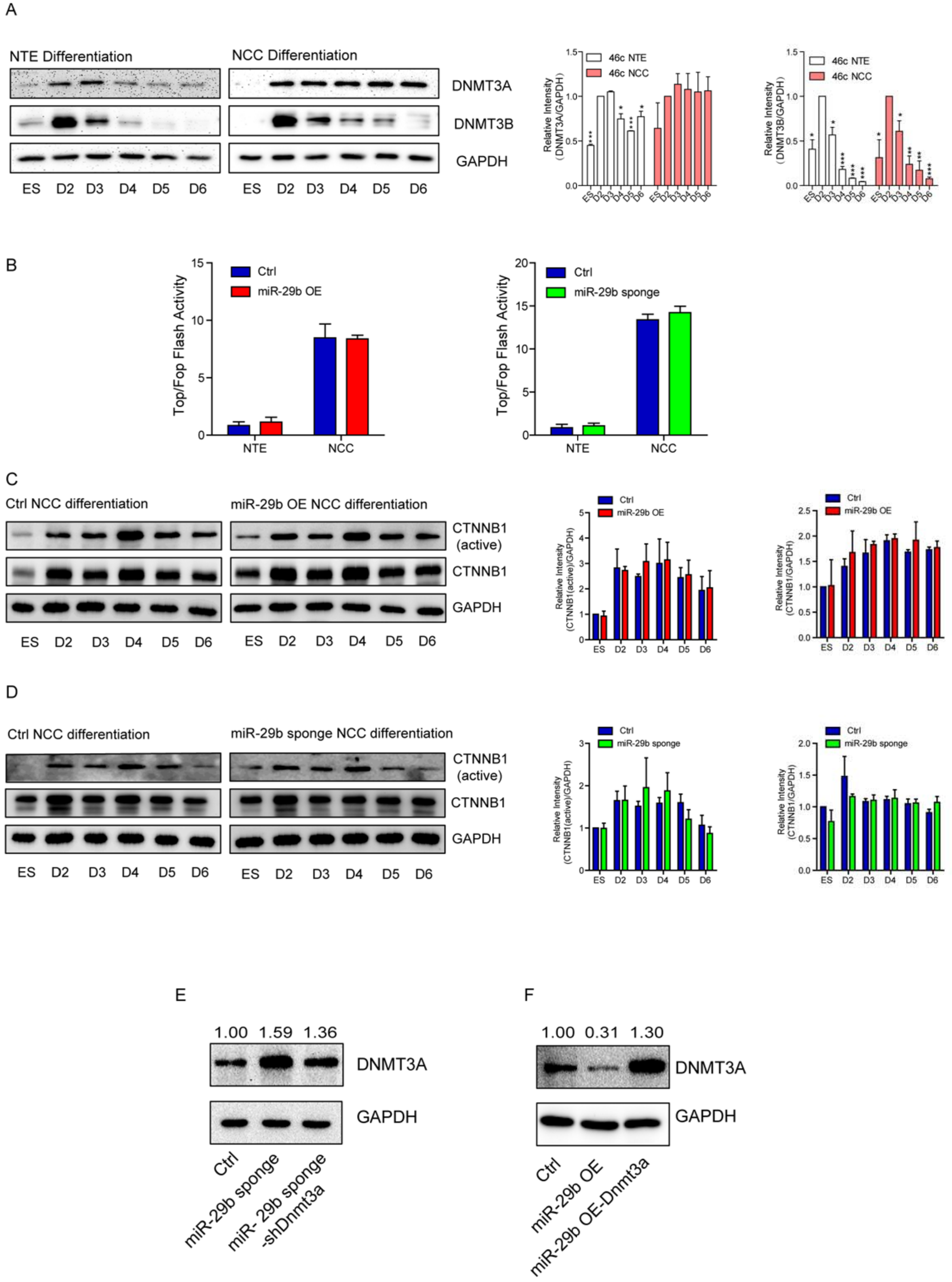
A
NTE Differentiation
NCC Differentiation
DNMT3A
DNMT3B
GAPDH
ES D2 D3 D4 D5 D6
46c NTE
46c NCC
Relative Intensity (DNMT3A/GAPDH)
Relative Intensity (DNMT3B/GAPDH)
B
Ctrl
miR-29b OE
miR-29b sponge
Top/Fop Flash Activity
NTE
NCC
C
Ctrl NCC differentiation
miR-29b OE NCC differentiation
CTNNB1 (active)
CTNNB1
GAPDH
Relative Intensity (CTNNB1(active)/GAPDH)
Relative Intensity (CTNNB1/GAPDH)
D
Ctrl NCC differentiation
miR-29b sponge NCC differentiation
E
1.00 1.59 1.36
DNMT3A
GAPDH
Ctrl
miR-29b sponge
miR- 29b sponge -shDnmt3a
F
1.00 0.31 1.30
DNMT3A
GAPDH
Ctrl
miR-29b OE
miR-29b OE-Dnmt3a

Figure S3

A B C

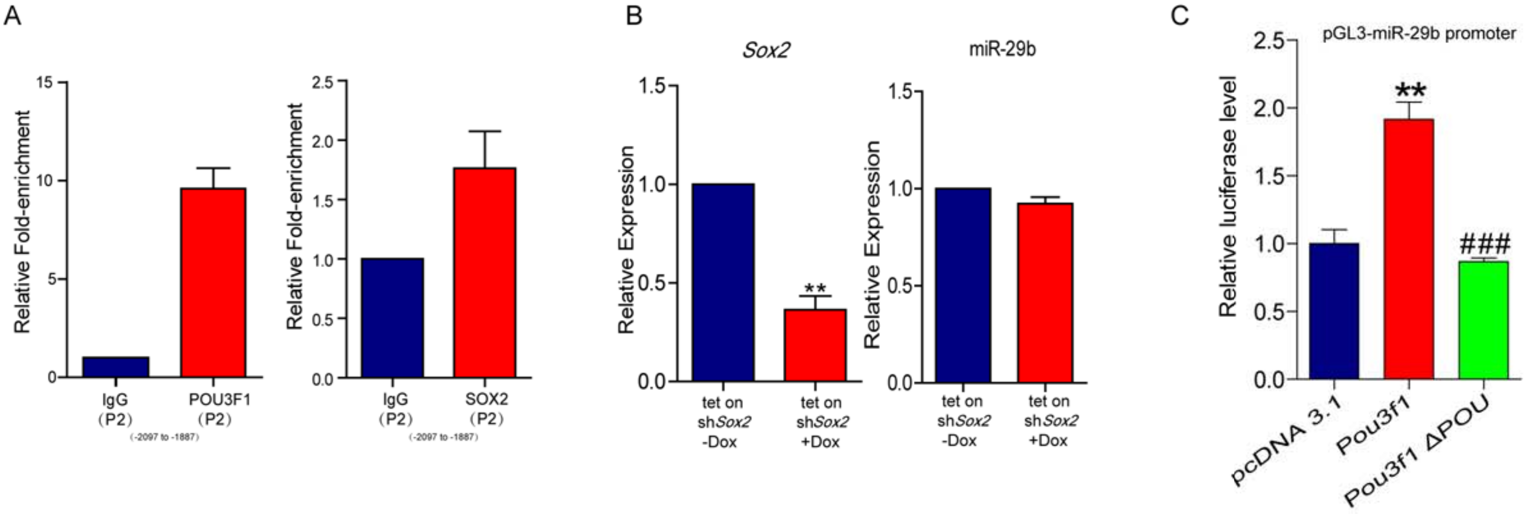

D

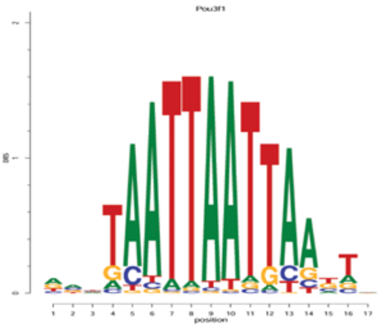

E

Sequence for POU3F1 binding based on the motif of POU3F1.(p value <0.001)

motif_alt_id	sequence_name	start	stop	strand	p-value	matched_sequence
NNNTAATTAATTARBDN	Pou3f1	-1540	-1524	-	0.000631	AATAAATAAATAAAATA
NNNTAATTAATTARBDN	Pou3f1	-1539	-1523	-	0.000414	AAATAAATAAATAAAAT
NNNTAATTAATTARBDN	Pou3f1	-1539	-1523	+	0.000866	attttatttatttattt
NNNTAATTAATTARBDN	Pou3f1	-1538	-1522	+	0.000966	ttttatttatttattta
NNNTAATTAATTARBDN	Pou3f1	-1536	-1520	-	0.000617	AATAAATAAATAAATAA
NNNTAATTAATTARBDN	Pou3f1	-1535	-1519	-	0.000163	AAATAAATAAATAAATA
NNNTAATTAATTARBDN	Pou3f1	-1535	-1519	+	0.000766	tatttatttatttattt
NNNTAATTAATTARBDN	Pou3f1	-1534	-1518	+	0.000644	atttatttatttatttt
NNNTAATTAATTARBDN	Pou3f1	-1210	-1194	-	0.000451	AGATAAAAAAATTAAAAA

F G

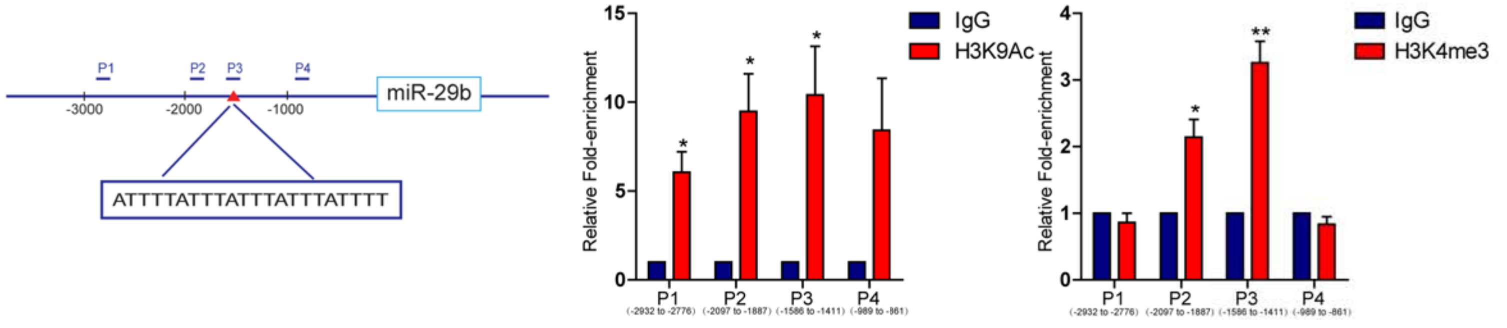

H I J

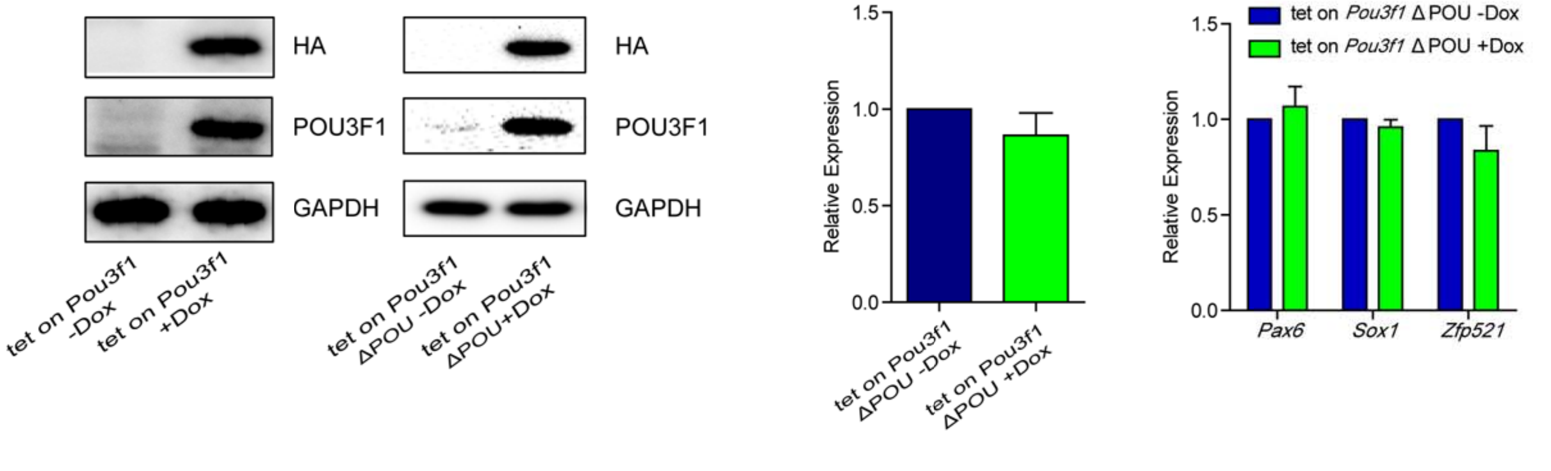

Figure S4

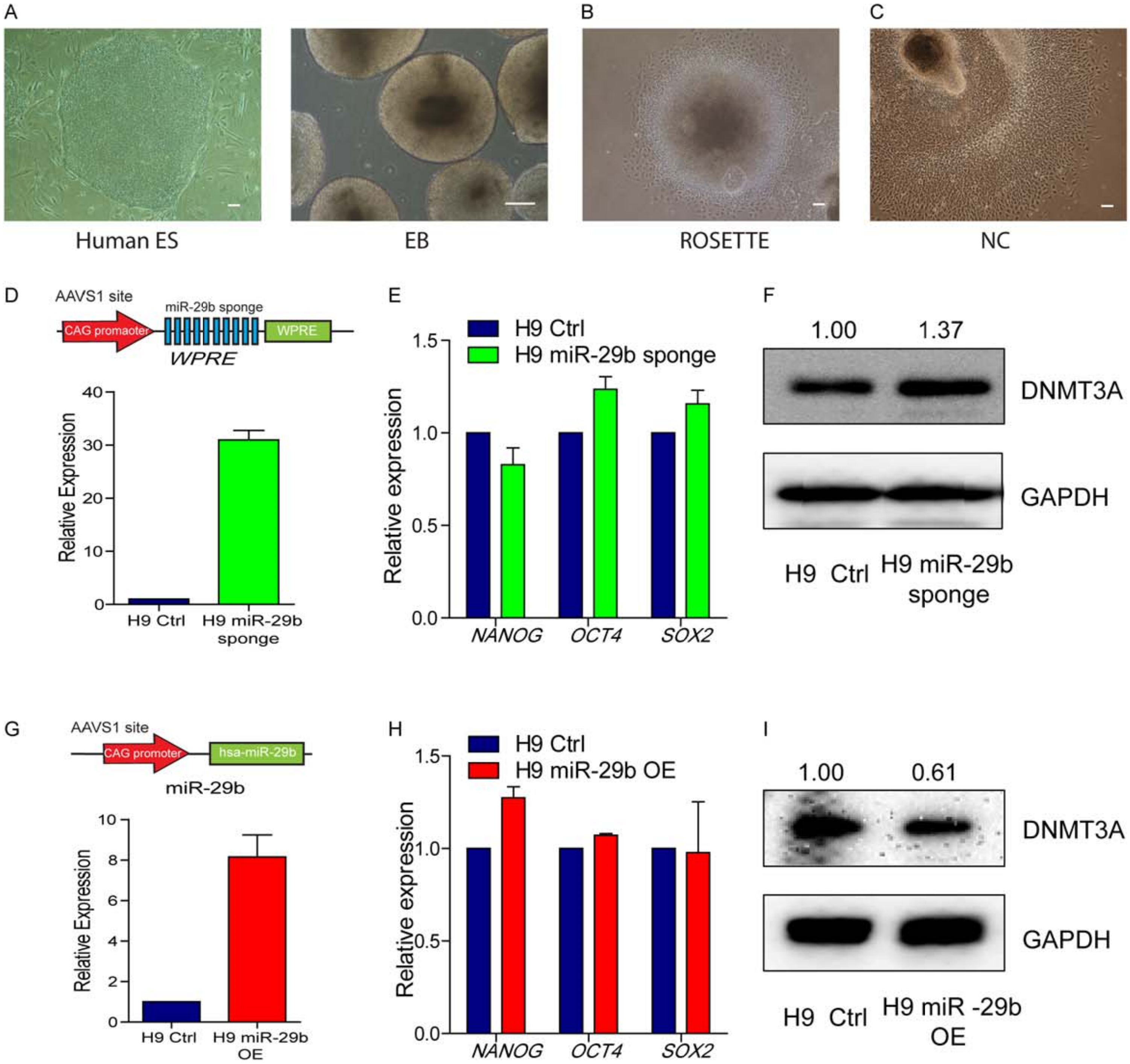
A
B
C
Human ES
EB
ROSETTE
NC
D
AAVS1 site
miR-29b sponge
CAG promaoter
WPRE
WPRE
Relative Expression
40
30
20
10
0
H9 Ctrl
H9 miR-29b sponge
E
H9 Ctrl
H9 miR-29b sponge
Relative expression
1.5
1.0
0.5
0.0
NANOG
OCT4
SOX2
F
1.00
1.37
DNMT3A
GAPDH
H9 Ctrl
H9 miR-29b sponge
G
AAVS1 site
CAG promoter
hsa-miR-29b
miR-29b
Relative Expression
10
8
6
4
2
0
H9 Ctrl
H9 miR-29b OE
H
H9 Ctrl
H9 miR-29b OE
Relative expression
1.5
1.0
0.5
0.0
NANOG
OCT4
SOX2
I
1.00
0.61
DNMT3A
GAPDH
H9 Ctrl
H9 miR -29b OE

Figure S1.

Q-PCR assay showed neither inhibition (A) or overexpression (B) of miR-29b substantially influences the expression of pluripotent genes in mouse ES cells.

Data are shown as means ± SEM of at least three independent experiments.

Figure S2.

(A) Western blotting showed the expression profiles of DNMT3A and DNMT3B during NTE and NCC differentiation respectively. GAPDH is the normalization control. The protein abundance of DNMT3A and DNMT3B was quantified with normalization by signals of GAPDH.

(B) TOP/FOP flash assay for D4 cells in ctrl, miR-29b OE cell line or ctrl, miR-29b sponge cell line during NTE and NCC differentiation respectively.

(C and D) Western blotting analyzed the protein levels of total and active CTNNB1 in ctrl, miR-29b OE cell line during NCC differentiation (C) or ctrl, miR-29b sponge cell line during NCC differentiation (D). GAPDH is the normalization control. The protein abundance of total and active CTNNB1 was quantified with normalization by signals of GAPDH.

(E) DNMT3A knock down efficiency in miR-29b sponge cell line, as verified by western blotting. GAPDH is the normalization control. The protein abundance of DNMT3A was quantified with normalization by signals of GAPDH.

(F) DNMT3A overexpression efficiency in miR-29b OE cell line, as verified by western blotting. GAPDH is the normalization control. The protein abundance of DNMT3A was quantified with normalization by signals of GAPDH.

Data are shown as means ± SEM of at least three independent experiments. Unpaired two-tailed Student's t test was used. *$p<0.05$, **$p<0.01$ versus the control or D2 protein level (A).

Figure S3.

(A) ChIP assay showed the enrichment of POU3f1, but not SOX2 at the upstream region of miR-29b.

(B) Q-PCR measured the expression levels of *Sox2* and miR-29b in tet on sh*Sox2* cell line with and without Dox.

(C) Luciferase reporter assay in 3T3 cells transfected control (pcDNA3.1), *Pou3f1* and *Pou3f1ΔPOU* respectively with pGL3-miR-29b promoter.

(D) Binding motif of POU3F1 based on the UniPROBE database.

(E) Analysis of matched sequence and position in the 3kb region upstream of miR-29b based on the motif of POU3F1.

(F) Schematics showing the position of primers (P1, P2, P3 and P4) for ChIP assay and predicated POU3F1 binding site in the 3kb region upstream of miR-29b (red delta).

(G) ChIP assays showed the enrichment of H3K9Ac and H3K4me3 in the upstream region of miR-29b.

(H) Overexpression efficiency of HA-POU3F1 (left) and HA-POU3F1ΔPOU (right) with the inducible system, as verified by western blotting.

(I) Overexpressing of *Pou3f1ΔPOU* didn't affect the expression level of miR-29b on D3 of NTE differentiation.

(J) Overexpression of *Pou3f1ΔPOU* didn't promote NTE differentiation as shown by Q-PCR on D5 of NTE differentiation.

Data are shown as means ± SEM of at least three independent experiments. Unpaired two-tailed Student's t test was used. *$p<0.05$, **$p<0.01$ versus the control, $^{###}p<0.001$ versus the *Pou3f1* group (C).

Figure S4.

(A) hESCs growing on MEF feeders and formed embryoid bodies (EBs) in suspension culture.

(B) Columnar epithelial cells at D10 organized into rosettes like structure in NTE differentiation.

(C) Stellate-morphology cells migrated out of the spheres after the EBs were attached to the Matrigel-coated dish.

(D) Diagram of insert a CAG promoter driving ten-copy miR-29b sponge into AAVS1

site and the expression level of miR-29b sponge verified by Q-PCR.

(E) Q-PCR assay showed inhibition of miR-29b did not influence the expression of pluripotent marker genes in human ES cells.

(F) Western blotting analyzed the expression level of DNMT3A in human ES after inhibiting miR-29b. The protein abundance of DNMT3A was quantified with normalization by signals of GAPDH.

(G) Diagram of insert a CAG promoter driving miR-29b cassette into AAVS1 site and miR-29b overexpression efficiency, as verified by the expression level of miR-29b through Q-PCR.

(H) Q-PCR assay showed overexpression of miR-29b did not influence the expression of pluripotent marker genes in human ES cells.

(I) Western blotting analyzed the expression level of DNMT3A in human ES after overexpressing miR-29b. The protein abundance of DNMT3A was quantified with normalization by signals of GAPDH.

Data are shown as means ± SEM of at least three independent experiments. Unpaired two-tailed Student's t test was used. Scale bars, 100 μm.

Supplemental Tables

Table S1. Primers used for vectors construction

miR-29b sponge (3 repeats)	AACACTGATTGTTATGGTGCTACCGAACACTGATTGTTATGGTGCTAGCGAACACTGATTGTTATGGTGCTA	F
	TAGCACCATAACAATCAGTGTTCGCTAGCACCATAACAATCAGTGTTCGGTAGCACCATAACAATCAGTGTT	R
Dnmt3a	CGGGATCCATGCCCTCCAGCGGCCCCGGGGA	F
	GCTCTAGATTACACACAAGCAAAATATTCCTTCAG	R
Dnmt3a-shRNA	CCGGGTGCAGAAACATCGAGGACATCTCGAGATGTCCTCGATGTTTCTGCACTTTTTG	F
	AATTCAAAAAGTGCAGAAACATCGAGGACATCTCGAGATGTCCTCGATGTTTCTGCAC	R
Lucferase-shRNA	CCGGTGAAACGATATGGGCTGAATACTCGAGTATTCAGCCCATATCGTTTCATTTTT	F
	AATTAAAAATGAAACGATATGGGCTGAATACTCGAGTATTCAGCCCATATCGTTTCA	R
HA-Pou3f1	CGGGATCCATGTACCCATACGATGTTCCAGATTACGCTGCCACCACCGCGCAGTATC	F
	GGAATTCTCACTGCACAGAGCCGGGCAGTG	R
Mmu-miR-29b Pri	CGCGGATCCCCTGAAATTAGGAACTATTGCACGGAC	F
	CGCGTCGACTAGCCTGTTTTAGACACTGGACACTTA	R
Hsa-miR-29b Pri	CGGGATCCCAGGCATGCTCTCCCATCAATAAC	F
	GCTCTAGAACTGCCATTTGTGATATATGCCACC	R
Pou3f1-shRNA	CCGGGCAGCGGAAGATCCAGAATCTCGAGATTCTGGATCTTCCGCTGCTTTTTG	F
	AATTCAAAAAGCAGCGGAAGATCCAGAATCTCGAGATTCTGGATCTTCCGCTGC	R
Sox2-shRNA	CCGGGCCCTGCAGTACAACTCCATGCTCGAGCATGGAGTTGTACTGCAGGGCTTTTTG	F
	AATTCAAAAAGCCCTGCAGTACAACTCCATGCTCGAGCATGGAGTTGTACTGCAGGGC	R
WPRE	GGAATTCAATCAACCTCTGGATTACAAAATTTGTG	F
	CCGCAATTGCAGGCGGGGAGGCGGCCCAAAGGGA	R
Mmu-miR-29b promoter	GGGGTACCTCCCGCGTGGAGGCCCTTGAATGGA	F
	CGACGCGTGAAGAAGCTTTGTCGTCTGTTTTTG	R
Mmu-miR-29b promoter Fragment-A	GGGGTACCTCCCGCGTGGAGGCCCTTGAATGGA	F
	CGACGCGTGCGGACTCTGGGGCCTTCCATGGCC	R
Mmu-miR-29b promoter Fragment-B	GGGGTACCACTTGTATGCATGTCCTGAGGGAAG	F
	CGACGCGTGATAATCAAACAAATTCCTGTATTTGTGC	R
Mmu-miR-29	GGGGTACCATTGAAAGTTAATGCCATGTAGTGAG	F

b promoter		
Fragment-C	CGACGCGTGAAGAAGCTTTGTCGTCTGTTTTTG	R

Table S2. Primer sets used in Q-PCR assays

Human & Mouse *Sox1*	GTTTTTTGTAGTTGTTACCGC	F
	GCATTTACAAGAAATAATAC	R
Human & mouse *GAPDH*	ATGACATCAAGAAGGTGGTG	F
	CATACCAGGAAATGAGCTTG	R
Human & Mouse *Nanog*	ATTCTTCCACCAGTCCCAAA	F
	ATCTGCTGGAGGCTGAGGTA	R
Human & Mouse *Pax6*	TCTTTGCTTGGGAAATCCG	F
	CTGCCCGTTCAACATCCTTAG	R
Human *OCT4*	ACATCAAAGCTCTGCAGAAAGAACT	F
	CTGAATACCTTCCCAAATAGAACCC	R
Human *P75*	GCAGAACAAGCAAGGAGCCA	F
	CCGCAGAGCCGTTGAGAAG	R
Human *SNAIL2*	TGTGACAAGGAATATGTGAGCC	F
	TGAGCCCTCAGATTTGACCTG	R
Human *SOX10*	CCTCACAGATCGCCTACACC	F
	CATATAGGAGAAGGCCGAGTAGA	R
Human *SOX2*	GCCCTGCAGTACAACTCCAT	F
	TGGAGTGGGAGGAAGAGGTA	R
Human *ZNF521*	TTCCGAGCAAGTGCAGAAAG	F
	AAGGTTCGAGAGCACACGTTG	R
Mouse *Fgf5*	AAAGTCAATGGCTCCCACGAA	F
	GGCACTTGCATGGAGTTTTCC	R
Mouse *Nestin*	CTGCAGGCCACTGAAAAGTT	F
	GACCCTGCTTCTCCTGCTC	R
Mouse *Oct4*	ACATGAAAGCCCTGCAGAAGGAGCT	F
	GAGAACGCCCAGGGTGAGCC	R
Mouse *P75*	ACCCGAGGCACCGCTGACAACCTCA	F
	GGCTACTGTAGAGGTTGCCATCACC	R
Mouse *Pou3f1*	AGTTCGCCAAGCAGTTCAAG	F
	TGGTCTGCGAGAACACGTTA	R
Mouse *Rex1*	GGAAGAAATGCTGAAGGTGGAGAC	F
	AGTCCCCATCCCCTTCAATAGC	R
Mouse *Snail2*	TGGTCAAGAAACATTTCAACGCC	F
	GGTGAGGATCTCTGGTTTTGGTA	R
Mouse *Sox10*	AAAGGACCATCCGGACTACAAGTA	F
	CTGCAGCTCTGTCTTTGGGGTG	R
Mouse *Sox2*	CAGGAGAACCCCAAGATGCACAA	F

	ATTAAGCTCCTGGGTCGCAAG	R
Mouse *Tubb3*	TAGACCCCAGCGGCAACTAT	F
	GTTCCAGGTTCCAAGTCCACC	R
Mouse *Zfp521*	GAGCGAAGAGGAGTTTTTGG	F
	AGTTCCAAGGTGGAGGTCAC	R

Table S3. Primers for ChIP assay

P1 (-2932 to -2776)	GAGATGGGAGGCACAAGGCAAAG	F
	GGTTAAATCAGGTAGTACATGAGATGAT	R
P2 (-2097 to -1887)	AGGGAGAAGGGAAGCACGGGCACA	F
	AGGAACAGGCTCACCATGAGAATAG	R
P3 (-1586 to -1411)	TGAACATTTTCTTTCGGTTTGGTG	F
	CAGGCTACAGACTGGGAATCTATTTC	R
P4 (-989 to -861)	ATGCCATGTAGTGAGCCCTGAAATT	F
	CCTTTATCCCTTCCTGCTCATTCTC	R

Table S4. Antibodies for immunostaining analysis and FACS

**Antibodies**	**Manufacture**	**Catalog**
Anti-P75	abcam	ab38335
Anti-PAX6	DSHB	PAX6
Anti-GFP	Proteintech	50430-2-AP
Anti-SOX1	R&D	AF3369
Anti-SOX2	R&D	MAB2018R
Anti-FOXD3	Santa Cruz	sc-133588
Anti-P75	Santa Cruz	sc-6188
Anti-SOX10	Santa Cruz	sc-17342
Donkey anti-Rabbit IgG 594	Life technologies	A21207
Donkey anti-Rabbit IgG 488	Life technologies	A21206
Donkey anti-Mouse IgG 594	Life technologies	A21203
Donkey anti-Mouse IgG 488	Life technologies	A21202
Donkey	Life technologies	A11058

anti-Goat IgG 594		
Donkey anti-Goat IgG 488	Life technologies	A11055
Donkey anti-Goat IgG 647	Life technologies	A21447
Ho.33342	Sigma	14533

Table S5. Antibodies for Western blotting analysis

Antibodies	Manufacture	Catalog
Anti-HA	Abcam	ab9110
Anti-POU3F1	Abcam	ab31766
Anti-CTNNB1	Abcam	Ab22656
Anti-active CTNNB1	Millipore	07-473
Anti-GAPDH	Bioworld	AP0063
Anti-DNMT3A	Cell Signaling Technology	3598
Anti-DNMT3B	Cell Signaling Technology	2161
Anti-Rabbit IgG HRP	Cell Signaling Technology	7074S
Anti-mouse IgG HRP	Cell Signaling Technology	7076S

Table S6. Antibodies for ChIP analysis

Antibodies	Manufacture	Catalog
Anti-SOX2	Abcam	ab59776
Anti-POU3F1	Santa Cruz Biotechnology	Sc-28593
Anti-H3K4me3	Millipore	07-473
Anti-H3K9Ac	Millipore	06-942
Rabbit IgG	Cell Signaling Technology	2729s

Supplemental Experimental procedures

Mouse ES cell (mESC) culture and differentiation

The mESC Sox1-GFP (46c，passages 12-25) was cultured on a feeder layer in Dulbecco’s Modified Eagle’s Medium (DMEM, GIBCO) supplemented with 15% fetal bovine serum (FBS, GIBCO), 2 mM L-glutamax (Invitrogen), 2 mM nonessential amino acids (NEAA, Invitrogen), 1 mM sodium pyruvate (Invitrogen), 0.1 mM β-mercaptoethanol (Sigma), and 20 ng/ml homemade LIF. For differentiation, mESCs were dissociated and suspended on 10-cm Petri dishes at a density of 50000 cells/mL with 10 mL in growth minimal essential medium (GMEM) (Gibco) supplemented with 8% knockout serum replacement (KOSR; Gibco), 2 mM L-glutamax, 2 mM NEAA, 1 mM sodium pyruvate and 0.1 mM β-mercaptoethanol. After 2 days of incubation, the cells aggregated to form embryoid bodies (EBs). The NTE differentiation was performed according to published protocols (Wang et al., 2011), the D2 EBs were then continuously cultured in neural differentiation medium consisting of DMEM/F12 (Invitrogen), N2 supplement (Invitrogen), B27 supplement (without vitamin A, Invitrogen), 2 mM L-glutamax, 2 mM NEAA, and chemically defined lipid concentrate (Invitrogen) for the next 4-6 days. The NCC differentiation was performed modified from published protocol (Minamino et al., 2015), the D2 EBs were cultured in neural differentiation medium supplemented with FGF2 (10 ng/ml, Sino Biological, CAT# 10014-HNAE-500) and BIO (0.6 μM, Selleck, CAT# S7198) for the next 4-6 days. After EBs attached to Matrigel-coated culture dishes and continue cultured in neural differentiation medium, the NCC migrated out of the spheres.

Human ESC (hESC) culture and differentiation

Human ESC H9 (WiCell Institute, Madison, WI, USA, passages 25-45) was cultured on irradiated mouse embryonic fibroblasts (MEFs) as described in the standard protocol (http://www.wicell.org). The NTE differentiation of hESCs was performed according to published protocols (Zhang et al., 2001; Zhang et al., 2010). Human ESCs were detached by incubating with Dispase and the colonies were

cultured in suspension as EBs in the hESC medium consisting of DMEM/F12 (GIBCO), 20% KOSR, 2 mM NEAA, 2 mM L-glutamax, and 0.1 mM β-Mercaptoethanol. On D4, the hESC medium was replaced with a neural differentiation medium consisting of DMEM/F12, N2 supplement, 2 mM NEAA and 2 μg/ml heparin (Sigma, Saint Louis, MO). On D7, the EBs were attached to the plastic or laminin-coated substrate and cultured in the neural differentiation medium and columnar neuroepithelia organized into rosette structures in 3-5 days. The NCC differentiation of hESCs was performed modified from published protocol (Liu et al., 2012; Menendez et al., 2013), the hESC colonies were cultured in the hESC medium to form EBs, on D4 the hESC medium was replaced with a neural differentiation medium, FGF2 (10 ng/ml) and BIO (0.4 μM) were added to the medium from D4 to D10. After the EBs were attached to the Matrigel-coated dishes on D8 and continue cultured in neural differentiation medium, the neural crest cells with a stellate morphology migrated out of the spheres in 2-3 days.

Gene overexpression and knockdown in ESCs

For the constitutive overexpression of genes in mESCs, miR-29b and miR-29b sponge were driven by the CAG promoter integrated into the Rosa26 locus through electroporation with engineered zinc finger nucleases, as previously described (Perez-Pinera et al., 2012). The mESCs with a CAG-*RBGpA* in Rosa26 locus were used as control. For the constitutive overexpression of genes in hESCs, miR-29b and the miR-29b sponge were driven by the CAG promoter integrated into the AAVS1 locus through electroporation with TALEN as previously described (Hockemeyer et al., 2011). The hESCs with a CAG-*RBGpA* in AAVS1 locus were used as control.

For the inducible overexpression system, advanced *rtTA* driven by the CAG promoter was integrated into the Rosa26 locus through electroporation with engineered zinc finger nucleases as previously described. A lentiviral backbone comprising the tetracycline response element (TRE) driving *HA-Pou3f1* and *HA-Pou3f1 ΔPOU*(missing amino acids 241–395) (Sock et al., 1996) were cloned and used for virus packaging. Medium containing viral particles was then added to the

rtTA ESC line for efficient infection. For *Dnmt3a* overexpression in miR-29b OE cells, a *Dnmt3a* CDS sequence was cloned into the pCAG-puroR lentiviral vector. For miR-29b sponge overexpression in tet on *Pou3f1* cells, miR-29b sponge sequence was cloned into the pCAG-puroR lentiviral vector. The CAG-RBGpA-puroR lentivirus was used as the control.

All primers used for gene cloning are listed in Table S1.

For *Dnmt3a* knockdown in miR-29b sponge cells, shRNA targeting *Dnmt3a* was cloned into the PLKO lentiviral vector as previously described. A shRNA targeting *Luciferase* (sh*Luc*) was also designed as a control (Yang et al., 2014). The shRNA targeting *Pou3f1* (Zhu et al., 2014) and *Sox2* were cloned into the Tet-on PLKO lentiviral vector. The shRNA target sequence is listed in Table S1. Lentivirus packaging and infection were performed as previously described (Yang et al., 2014).

RNA extraction, cDNA synthesis and Q-PCR analysis

Total RNA was extracted using RNAiso Plus (TaKaRa). Reverse transcription of 500 ng RNA was performed using a PrimeScript RT reagent kit (TaKaRa). Quantitative PCR (Q-PCR) was performed using a SYBR Premix Ex TaqTM Kit (TaKaRa). Gene expression was normalized to *Gapdh*. Q-PCR primers are listed in Table S2.

For quantitative miRNA analyses, reverse transcription of 500 ng RNA was performed using a miRcute miRNA First-Strand cDNA Synthesis Kit (Tiangen). Q-PCR was performed using a miRcute miRNA qPCR Detection Kit (Tiangen). The miRNA expression was normalized to U6.

Immunofluorescence staining

Immunofluorescence staining was performed as previously described (Xi et al., 2012). For mESC-derived NTE and NCC EBs staining, the cultured EBs were harvested and fixed with 4% paraformaldehyde at 4℃ for 15 min and dehydrated with a sucrose gradient before frozen sectioning. For mESC-derived NCC staining, the NCC EBs on D8 were plated onto 12-mm coverslips coated with poly-ornithine and

2% (v/v) Matrigel (BD Biosciences) for 2~4 days and fixed with 4% paraformaldehyde at 4℃ for 15 min. For hESC-derived NTE and NCC staining, the EBs were plated onto 12-mm coverslips coated with poly-ornithine and 2% (v/v) Matrigel for 3~5 days and fixed with 4% paraformaldehyde at 4℃ for 15 min.

The fixed frozen section slides and coverslip cultures were washed three times with PBS and blocked in PBS/10% donkey serum/0.1% Triton X-100 for 1 h at room temperature. Then, the samples were stained with a primary antibody overnight at 4℃, washed three times with PBS, and incubated with a secondary antibody/HO.33342 for 1 h at room temperature. Immunofluorescence images were captured by fluorescence microscopy (Nikon ECLIPSE Ti-S). Quantitative analysis was performed using ImageJ (https://imagej.nih.gov/ij/) software. The antibodies are listed in Table S4.

Western blotting

The cells were lysed in RIPA buffer. The protein concentration was detected by a BCA assay kit (Thermo). Equal amounts of cell lysates were loaded into a gel, blotted onto a PVDF membrane and probed with primary antibodies. GAPDH was used as a loading control. The antibodies used for western blotting are listed in Table S5. The membranes were scanned with an ImageQuant LAS 4000 System (GE Healthcare Life Sciences) and quantitatively analyzed with AlphaView (http://www.proteinsimple.com) software.

Luciferase assays

The luciferase assay was previously described (Guo et al., 2013). For the miR-29b promoter luciferase assay, miR-29b promoter sequence was cloned into the pGL3-basic vector (Promega). The primers used for miR-29b promoter gene cloning are listed in Table S1. The deletion of a 100 bp region in fragment-B was performed by Hieff Clone™ Plus Multi One Step Cloning Kit (Yeasen, CAT# 10912ES10)

NIH 3T3 cells grown in 24-well plates were transfected with 100 ng each of pcDNA3.1, pcDNA-*Sox1*, pcDNA-*Sox2*, pcDNA-*Pax6*, pcDNA-*Zfp521*,

pcDNA-*Zic1*, pcDNA-*Pou3f1* or pcDNA-*Pou3f1 △POU*, 200 ng of a pGL3-basic-29b promoter vector(full length or fragment), and 10 ng of a control Renilla luciferase vector (pRL-Tk; Promega) using X-tremeGENE HP DNA Transfection Reagen (Roche). After 48 h, the cells were lysed in lysis buffer. Firefly and Renilla luciferase activities were assayed using a SpectraMax M5 plate reader (Molecular Device). The firefly luciferase signal was normalized against the Renilla signal, and pcDNA3.1 was used as a negative control.

For the TOPFlash/FOPFlash assays, the D2 EBs cultured in 6 cm Petri Dish were co-transfected with 2 μg of TOPFlash or FOPFlash plasmid (M50 Super 8x TOPFlash and M51 Super 8x FOPFlash was a gift from Randall Moon, Addgene plasmid # 12456 and 12457) and 20 ng of a control Renilla luciferase vector using X-tremeGENE HP DNA Transfection Reagen (Roche), then continue cultured for NTE and NCC differentiation, cells were lysed in lysis buffer 48h post transfection. Firefly and Renilla luciferase activities were assayed using a SpectraMax M5 plate reader (Molecular Device). The firefly luciferase signal was normalized against the Renilla signal.

Chromatin immunoprecipitation (ChIP)

The ChIP assay was previously described (Wei et al., 2015). ChIP was performed with 4 μg antibody according to a previously described protocol. Normal rabbit IgG (Millipore) was used as a negative control. Immunoprecipitated DNA was used as a template for Q-PCR analysis with primers against miR-29b promoter regions. PCR reactions were performed using a SYBR Premix Ex TaqTM Kit (TaKaRa). The primers used for ChIP-Q-PCR are listed in Table S3. The antibodies are listed in Table S6.

Fluorescence-activated cell sorting analysis (FACS)

FACS was performed as previously described (Wang et al., 2013). For endogenous GFP analysis, cultured EBs were trypsinized into single cells, washed once in PBS and strained through a fine-mesh sieve prior to sorting using a FACSCalibur (BD

Biosciences). The undifferentiated mESCs were used as the negative control.

For P75 staining, the cells were harvested using 0.05% trypsin-EDTA at 37℃ for 2 min, fixed in 4% paraformaldehyde, washed twice in PBS, and incubated in 100 μL of 1% donkey serum in PBS containing a goat anti-P75 antibody (1:200 dilution; Santa Cruz Technology) for 1 h and washed three times in PBS. The cells were resuspended in 100 μL of 1% donkey serum in PBS containing an Alexa Fluor 647-labeled donkey anti-goat IgG antibody (1:1000 dilution) and incubated for 30 min at room temperature. The cells were washed three times and resuspended in 500 μL of PBS. The proportion of P75–positive cells was analyzed by a FACSCalibur (BD Biosciences). The NIH 3T3 cells were used as the negative control. The FACS data were analyzed using FlowJo (http://www.flowjo.com) software.

Statistical analyses

All statistical data were presented as the mean±SEM of at least three independent experiments. Statistical significance was calculated according to unpaired two-tailed Student's t tests using GraphPad Prism Software. $P<0.05$ was considered statistically significant. * and $^{\#}$ indicates $P< 0.05$, ** and $^{\#\#}$ indicates $P< 0.01$, and *** and $^{\#\#\#}$ indicates $P< 0.001$.

Supplemental Reference

Guo, X., Liu, Q., Wang, G., Zhu, S., Gao, L., Hong, W., Chen, Y., Wu, M., Liu, H., Jiang, C., et al. (2013). MicroRNA-29b is a novel mediator of Sox2 function in the regulation of somatic cell reprogramming. Cell Res 23, 142-156.

Hockemeyer, D., Wang, H., Kiani, S., Lai, C.S., Gao, Q., Cassady, J.P., Cost, G.J., Zhang, L., Santiago, Y., Miller, J.C., et al. (2011). Genetic engineering of human pluripotent cells using TALE nucleases. Nat Biotechnol 29, 731-734.

Liu, Q., Spusta, S.C., Mi, R., Lassiter, R.N., Stark, M.R., Hoke, A., Rao, M.S., and Zeng, X. (2012). Human neural crest stem cells derived from human ESCs and induced pluripotent stem cells: induction, maintenance, and differentiation into functional schwann cells. Stem Cells Transl Med

1, 266-278.

Menendez, L., Kulik, M.J., Page, A.T., Park, S.S., Lauderdale, J.D., Cunningham, M.L., and Dalton, S. (2013). Directed differentiation of human pluripotent cells to neural crest stem cells. Nat Protoc 8, 203-212.

Minamino, Y., Ohnishi, Y., Kakudo, K., and Nozaki, M. (2015). Isolation and propagation of neural crest stem cells from mouse embryonic stem cells via cranial neurospheres. Stem cells and development 24, 172-181.

Perez-Pinera, P., Ousterout, D.G., Brown, M.T., and Gersbach, C.A. (2012). Gene targeting to the ROSA26 locus directed by engineered zinc finger nucleases. Nucleic acids research 40, 3741-3752.

Sock, E., Enderich, J., Rosenfeld, M.G., and Wegner, M. (1996). Identification of the nuclear localization signal of the POU domain protein Tst-1/Oct6. J Biol Chem 271, 17512-17518.

Wang, G., Guo, X., Hong, W., Liu, Q., Wei, T., Lu, C., Gao, L., Ye, D., Zhou, Y., Chen, J., et al. (2013). Critical regulation of miR-200/ZEB2 pathway in Oct4/Sox2-induced mesenchymal-to-epithelial transition and induced pluripotent stem cell generation. Proc Natl Acad Sci U S A 110, 2858-2863.

Wang, Z.B., Boisvert, E., Zhang, X., Guo, M., Fashoyin, A., Du, Z.W., Zhang, S.C., and Li, X.J. (2011). Fezf2 regulates telencephalic precursor differentiation from mouse embryonic stem cells. Cerebral cortex 21, 2177-2186.

Wei, T., Chen, W., Wang, X., Zhang, M., Chen, J., Zhu, S., Chen, L., Yang, D., Wang, G., Jia, W., et al. (2015). An HDAC2-TET1 switch at distinct chromatin regions significantly promotes the maturation of pre-iPS to iPS cells. Nucleic Acids Res 43, 5409-5422.

Xi, J., Liu, Y., Liu, H., Chen, H., Emborg, M.E., and Zhang, S.C. (2012). Specification of midbrain dopamine neurons from primate pluripotent stem cells. Stem Cells 30, 1655-1663.

Yang, D., Wang, G., Zhu, S., Liu, Q., Wei, T., Leng, Y., Duan, T., and Kang, J. (2014). MiR-495 suppresses mesendoderm differentiation of mouse embryonic stem cells via the direct targeting of Dnmt3a. Stem Cell Res 12, 550-561.

Zhang, S.C., Wernig, M., Duncan, I.D., Brustle, O., and Thomson, J.A. (2001). In vitro differentiation of transplantable neural precursors from human embryonic stem cells. Nat Biotechnol 19, 1129-1133.

Zhang, X., Huang, C.T., Chen, J., Pankratz, M.T., Xi, J., Li, J., Yang, Y., Lavaute, T.M., Li, X.J., Ayala,

M., et al. (2010). Pax6 is a human neuroectoderm cell fate determinant. Cell Stem Cell 7, 90-100.

Zhu, Q., Song, L., Peng, G., Sun, N., Chen, J., Zhang, T., Sheng, N., Tang, W., Qian, C., Qiao, Y., et al. (2014). The transcription factor Pou3f1 promotes neural fate commitment via activation of neural lineage genes and inhibition of external signaling pathways. eLife 3.
